# fNIRS, EEG, ECG, and GSR reveal an effect of complex, dynamically changing environments on cognitive load, affective state, and performance, but not physiological stress

**DOI:** 10.3389/fnhum.2025.1459653

**Published:** 2025-06-02

**Authors:** Henrikke Dybvik, Christian Kuster Erichsen, Chris Snider, Martin Steinert

**Affiliations:** ^1^School of Electrical, Electronic and Mechanical Engineering, University of Bristol, Bristol, United Kingdom; ^2^TrollLABS, Department of Mechanical and Industrial Engineering, Norwegian University of Science and Technology, Trondheim, Norway; ^3^SCANCOR, Graduate School of Education, Stanford University, Palo Alto, CA, United States

**Keywords:** human–machine interaction, workload, physiology sensors, neuroimaging, multimodal, affective state, Tetris

## Abstract

This study used functional near-infrared spectroscopy (fNIRS), electroencephalography (EEG), electrocardiography (ECG), electrodermal activity (EDA), performance, and subjective self-reports to investigate cognitive load and stress in a complex, dynamically changing environment. A total of 30 participants (*N* = 30) were assigned to three Tetris gameplays: Easy and Hard had constant difficulties, and Ramp started at a low difficulty level before successively ramping up to a very high difficulty level. Participants performed significantly better in Easy, followed by Ramp and Hard. In general, increased workload resulted in increased cognitive load and stress, but only up to a certain threshold, after which fNIRS activation reduced, possibly due to mental fatigue or disengagement. Furthermore, we found a temporal effect of workload in the constant workload conditions, evidenced by increased fNIRS activation (HbO increase and HbR decrease), and mental fatigue measured by EEG (Delta power increase). Despite significant differences in cognitive load, we found little between-condition differences in physiological stress response as measured by ECG and EDA. At the same time, Easy yielded significantly higher participant ratings of valence, enjoyment, workload acceptability, and subjective performance, compared to Hard, indicating differing affective states. The combination of undistinguishable physiological stress and varying affective states suggests that participants experienced more of a state of eustress in Easy and distress in Hard conditions.

## 1 Introduction

Understanding workload is critically essential in complex, high-risk dynamic environments, such as nuclear power plant control rooms, operating aircrafts, air traffic control towers, ship bridges, and shore control centers for remote ship operation (Causse et al., 2017; Hart and Wickens, 1990; Parent et al., 2019; Wulvik et al., 2019). An imbalanced workload adversely affects performance, and any errors may be accompanied by serious financial and fatal consequences (Causse et al., 2017; Hart and Wickens, 1990; Parent et al., 2019). High cognitive workload can induce cognitive tunneling, difficulties adapting to the situation, fatigue, and cognitive overload, which not only reduces performance but also increases human errors (Aghajani et al., 2017; Hart and Wickens, 1990; Parent et al., 2019). Low cognitive workload is also associated with increased risk of human errors, due to boredom, drowsiness, vigilance decrement, or lapses of attention (Hart and Wickens, 1990; Parent et al., 2019). Moreover, the acute psychological stress associated with these tasks impairs attention, memory, and decision-making, raising the likelihood of human error (Arnsten, 2009; Parent et al., 2019; Schoofs et al., 2009). In shipping for example, human errors are one of the most contributory factors to accidents (Fan et al., 2020; Hetherington et al., 2006; Weng et al., 2019). It is widely accepted that the majority of marine casualties are associated with human errors, with estimates ranging from 65% to 96%, depending on the type of accident and research method (Fan et al., 2020; Hetherington et al., 2006; Weng et al., 2019).

Today, many aspects of operation have moved into the control room, and human operators rely evermore on automated and autonomous systems (Hamann and Carstengerdes, 2022; Parent et al., 2019; Veitch et al., 2022; Wulvik et al., 2019). The maritime industry is transitioning toward remote and autonomous vessels that are navigated and monitored from an onshore control center, where operators will be responsible for more than one vessel (Veitch et al., 2022; Zhang et al., 2020). The transition is accompanied by highly automated and autonomous systems, intended to support decision-making and control, which, in turn, are designed to improve safety and efficiency (Hamann and Carstengerdes, 2022; Parent et al., 2019; Veitch et al., 2022). However, this is not always the case (Pazouki et al., 2018; Zhang et al., 2020). The paradoxical challenge of human–automation interaction is that with increasing automation, operators must keep track of growing numbers of systems and information magnitude, which could lead to data overload (Woods et al., 2002; Wulvik et al., 2019)—a situation that represents and potentially leads to cognitive overload. At the same time, operators are mainly monitoring systems and not actively controlling them (Bainbridge, 1983; Wulvik et al., 2019), representing cognitive underload, which could lead to boredom, fatigue, and vigilance decrements (Ahn and Jun, 2017; Parent et al., 2019; Wulvik et al., 2019). Apparently, the modern operating room is one where operators must juggle cognitive overload and underload and where failing to detect abnormalities could have life-threatening consequences. Adaptive systems accounting for day-to-day changes in human operators’ mental and physical state have long been proposed (Hamann and Carstengerdes, 2022; Parent et al., 2019). Adaptive systems continuously monitoring human operators cognitive load and stress could respond with suitable changes in e.g., which information is presented, the nature and modality of the human-machine interaction, or take urgent reactive measures in critical situations (Hamann and Carstengerdes, 2022; Parent et al., 2019).

While research agrees that human mental and physical state should be an integral part of interface and interaction designs in human–automation interaction (Parent et al., 2019; Veitch et al., 2022; Wulvik et al., 2019), the question of how this should be done remains unclear (Veitch et al., 2022). Moreover, we lack a comprehensive framework for investigating how humans function in and adapt to constantly changing environments (Kim et al., 2018). This situation highlights the importance of undertaking research aimed at understanding humans’ cognitive load and stress in complex, dynamic environments where potential critical situations could arise, warranting further research.

This study investigates cognitive load and stress in complex, dynamically changing environments, by dual-tasking human participants with a Tetris gameplay and an auditory reaction task (ART), while measuring functional near-infrared spectroscopy (fNIRS), electroencephalography (EEG), electrocardiogram (ECG), electrodermal activity (EDA), performance, and subjective self-reports.

We adopt a human-centered definition of the construct workload, considering workload or cognitive load as emerging from the interactions between task demands, context, the operator’s skills and cognitive resources, behavior, perceptions, and affective state (Causse et al., 2017; Hart and Staveland, 1988; Sheridan and Stassen, 1979; Wulvik et al., 2019; Xie and Salvendy, 2000). Here, cognitive load can be considered as a function of effort, which accounts for the capabilities (skills), motivation, and current-day state of the operator, required to maintain a given level of task performance (Cooper and Harper, 1969; Wulvik et al., 2019). Affect can be defined as variables that may influence behavior (Balters and Steinert, 2017). Affective state (or emotional state) can further be considered as a combination of multiple dimensions. We adopt the circumplex model of affect, which considers two dimensions: arousal (ranging from arousal to sleepiness) and valence (ranging from pleasure to displeasure) (Russel et al., 1989; Russell, 1980). We define mental state as the individual’s interpretation and manifestation of the concept of affective state. In this context, we define stress as something acute, as a state in which the sympathetic nervous system (SNS) is overactivated (Kim et al., 2018). Stress may further be defined as the body’s response to a stressing/stressful stimulus (allostasis) to maintain a state of stability (homeostasis) (Kim et al., 2018; Kupriyanov and Zhdanov, 2014; Selye, 1976). As such, stress may occur when posed physiological and mental demands are not adequately fulfilled by the parasympathetic nervous system (PNS) (Kim et al., 2018). However, all stress reactions are not equal (Szabo et al., 2012). Individual differences in subjective perceptions and emotional reactions give rise to distinguishing between “negative” stress, also known as “distress,” and “positive” stress, also known as “eustress” (Selye, 1976; Szabo et al., 2012). Distress is a stress response initiated by negative, unpleasant stressors, and “eustress” is a stress response triggered by positive emotions (Selye, 1976; Szabo et al., 2012). Distress could cause acute or chronic physical, psychological, and behavioral impairment (Kim et al., 2018) and it is often what is meant when the term “stress” is used in everyday life. Eustress could be considered a positive cognitive response to a stressor, possibly producing a positive effect (Kupriyanov and Zhdanov, 2014; Selye, 1976).

Workload is commonly manipulated by variations of the n-back among other standardized tasks (Aghajani et al., 2017; Meidenbauer et al., 2021; Owen et al., 2005). However, because such tasks are simple, most often containing only one element, they may not be used as an accurate representation of complex, dynamic work environments. We argue that such tasks differ too much, and that results from such studies cannot be generalized to complex, dynamic environments. Context-specific tasks, such as operational or navigational tasks employed with specialized simulation software, in e.g. aviation (Causse et al., 2017; Hamann and Carstengerdes, 2022) or ship navigation (Pazouki et al., 2018; Wulvik et al., 2019), are also common. Simulation software’s inherent limitation is that it requires participants to possess domain knowledge, be familiar with the software, or undergo extensive training, which could make recruitment difficult or necessitate time-consuming participant training. A good alternative is to use a task already familiar to participants or that is easily learned. Our goal was to create a complex, dynamic environment that could be representative of e.g., ship operation and aviation scenarios, that did not require specialized participant knowledge or skills, or high-end simulators.

Tetris selectively taxes visuospatial working memory (WM), and Tetris performance has a moderate positive correlation with standardized tests of visuospatial WM (Lau-Zhu et al., 2017). Improvements in spatial ability, mental rotation, and selective visual attention have been observed following cognitive training using Tetris (Lau-Zhu et al., 2017). High Tetris proficiency requires efficient deployment of working memory, mental rotation, strategic planning, prediction, manual dexterity, and more (Lindstedt and Gray, 2015). Tetris is a complex task in comparison to many standardized tasks for testing cognition (e.g., n-back), where players must respond to immediate time pressure, execute chains of manual commands while simultaneously planning for upcoming tetrominos (Lindstedt and Gray, 2015). As a tool for studying cognition and behavior, Tetris provides a dynamic task environment, with time-pressed decision-making, while preserving parametric control of workload and providing performance measures of high sensitivity (such as total score and level of play) (Lindstedt and Gray, 2015; Mallick et al., 2016). N-back demands working memory capacity because it requires continuous monitoring and updating of information (Owen et al., 2005), similar to Tetris. Similarly, as n-back may be stepwise increased to manipulate workload, Tetris levels also exhibit stepwise increase. We used Tetris to study cognitive load as its characteristics better represent the complex, dynamic environments we are interested in. Similar to other research (Mallick et al., 2016), we incorporate a secondary auditory task.

Cognitive load and stress may be measured through neuroimaging modalities, such as fNIRS and EEG, physiology sensors, such as ECG and galvanic skin response (GSR), along with behavioral measures, e.g., performance and reaction time, and subjective measures. Neuroimaging and physiology sensors are considered essential for enabling real-time monitoring of operators’ cognitive load (Antonenko et al., 2010; Borghini et al., 2014; Hamann and Carstengerdes, 2022).

fNIRS measures cortical brain activity optically, deriving concentration measures of oxygenated (HbO) and deoxygenated (HbR) hemoglobin in specific brain regions (Ferrari and Quaresima, 2012). fNIRS, which has been validated against neuroscience gold-standard functional magnetic resonance imaging (fMRI) (Cui et al., 2011; Ferrari and Quaresima, 2012; Huppert et al., 2006). Activation in the prefrontal cortex is often used as a measure of cognitive load. fNIRS is sensitive to changes in cognitive state and task load (Fishburn et al., 2014), in both classical working memory tasks and more contextual, operational tasks. Cortical activation is greater during lower workloads compared to higher workloads, as measured by HbO increase and HbR decrease, particularly in the right prefrontal cortex for a visuospatial working memory task (Baker et al., 2018), in bilateral dorsolateral prefrontal cortex (dlPFC), with the strongest activation in the left dlPFC for a letter n-back task (Fishburn et al., 2014), in frontal and parietal regions for a word n-back (Meidenbauer et al., 2021), and in the right dlPFC in a contextual aviation task (Hamann and Carstengerdes, 2022). When workload reaches a certain upper threshold, activation decreases compared to lower workloads (Baker et al., 2018; Hamann and Carstengerdes, 2022; Meidenbauer et al., 2021) suggesting participants cognitively disengage due to a lack of performance, or failing to recruit necessary cognitive resources (Meidenbauer et al., 2021). Moreover, brain activation is not necessarily linked to performance, but indicates differing individual neural efficiencies and an effect of expertise (Causse et al., 2017).

EEG measures electrical brain activity. EEG spectral power bands may be used as a continuous measure of cognitive load (Antonenko et al., 2010; Chikhi et al., 2022) as the relation between EEG spectral bands (in particular, Delta, Theta, Alpha, and Beta) and workload has been extensively studied (Antonenko et al., 2010; Borghini et al., 2014; Chikhi et al., 2022; Hamann and Carstengerdes, 2022). With increasing task difficulty and thus cognitive load Theta power increases, particularly in frontal brain regions, while Alpha power decreases in frontal, central, and parietal brain regions (Antonenko et al., 2010; Borghini et al., 2014; Chikhi et al., 2022; Hamann and Carstengerdes, 2022). Results for Beta power are mixed with literature reporting both Beta power decreases in parietal regions (Hamann and Carstengerdes, 2022) and Beta power increases with increasing cognitive load (Chikhi et al., 2022). The relationship between cognitive load and mental fatigue is crucial in certain workload scenarios. With increasing fatigue, Delta power increases (Borghini et al., 2014), Theta power increases in frontal and parietal brain regions (Borghini et al., 2014; Hamann and Carstengerdes, 2022), while Alpha power increases in frontal, occipital (Borghini et al., 2014), and parietal regions (Hamann and Carstengerdes, 2022). Again, literature reports mixed results for Beta power, reporting overall Beta power decreases (Borghini et al., 2014) and parietal Beta power increases with increasing mental fatigue (Hamann and Carstengerdes, 2022).

The autonomic nervous system (ANS) consists of the sympathetic nervous system (SNS) and the parasympathetic nervous system (PNS). The SNS and PNS work antagonistically to regulate physiological autonomic function (Appelhans and Luecken, 2006; Pham et al., 2021). The SNS is a quickly responding system, well-known as the fight-or-flight mechanism, generally activating in response to stimuli causing mental states of high arousal, and thus, SNS dominates during elevated activity and stressful states (Ishaque et al., 2021; Kim et al., 2018; Pham et al., 2021). Complementary, the PNS is a “relaxed response” system, predominating in quiet and relaxing states, known as the rest-and-digest mechanism, which relaxes the heart and lowers stress (Pham et al., 2021). The SNS and PNS work antagonistically together regulating physiological autonomic function (Appelhans and Luecken, 2006; Ishaque et al., 2021; Pham et al., 2021). Changes in sympathetic and parasympathetic activity manifest in various physiological phenomena, e.g., cardiac function, electrodermal activity, respiration, brain activity, etc. (Balters and Steinert, 2017).

ANS and PNS change cardiac activity, changing heart rate (HR) and heart rate variability (HRV). HR and HRV thus provide a measure of sympathetic and parasympathetic ANS function (Kim et al., 2018; Tarvainen et al., 2014). A range of variables can be derived to measure HRV (Pham et al., 2021). In this study, we focus our analysis on time- and frequency-domain derived variables only. Time-domain measures assess the variability in HR. In general, SNS tends to increase heart rate (HR) and decrease HRV, while PNS decreases HR and increases HRV (Ishaque et al., 2021; Kim et al., 2018; Pham et al., 2021; Tarvainen et al., 2014). Frequency-domain measures better assess specific components of HR (Pham et al., 2021). The HRV power spectrum can be meaningfully divided into four bands: ultra-low frequency (ULF ≤ 0.003 Hz), very low frequency (VLF; 0.0033–0.04 Hz), low frequency (LF; 0.04–0.15 Hz), and high frequency (HF; 0.15–0.4 Hz) (Malik et al., 1996). In controlled conditions, HF changes are modulated by PNS (Pham et al., 2021; Tarvainen et al., 2014), while LF changes are modulated by both PNS and ANS (Kim et al., 2018; Pham et al., 2021; Tarvainen et al., 2014). The normalized frequency components of LF and HF represent sympathetic and parasympathetic activity, respectively (Malik et al., 1996). The LF to HF power ratio measures the relative contributions of SNS to PNS activity (Shaffer and Ginsberg, 2017), increase in the LF to HF ratio is associated with psychological stress (Kim et al., 2018). Low PNS may be characterized by a decrease in the HF band and HF peak and an increase in the LF band and LF peak (Kim et al., 2018; Wulvik et al., 2019).

EDA is influenced by ANS activity only (Boucsein, 2012). Skin conductance (SC) is characterized by (a) slowly varying tonic activity, SC level (SCL), and (b) fast varying phasic activity, SC response (SCR). Tonic activity varies over minutes rather than seconds, and is related to continuous stimuli, e.g., performing a task (Benedek and Kaernbach, 2010; Boucsein, 2012). Phasic activity varies over seconds, is characterized by a steep incline to peak and a slow decline to baseline, and occurs in response to almost any stimulus that is novel, unexpected, or potentially important (Benedek and Kaernbach, 2010; Boucsein, 2012). Phasic activity may thus reflect both stimulus-specific responses and non-specific responses (Benedek and Kaernbach, 2010).

The present study uses fNIRS, EEG, ECG, EDA, performance, and subjective self-reports to assess cognitive load and stress in participants assigned with a primary task, a Tetris gameplay at different difficulties, and a secondary task, an ART. This article aims to investigate the relationship between workload, performance, and the human mental and physical state as measured by neuroimaging and physiology sensors. We set out to understand neurophysiology in complex, dynamically changing environments, and understand if and how well each tool can discern different workloads.

## 2 Materials and methods

### 2.1 Experiment design

#### 2.1.1 Stimuli: a modified Tetris gameplay

The experiment involved a modified version of the computer game Tetris® (Ros, 2024; Erichsen, 2020). Tetris is played on a 10 × 20 cell grid (the game space) where differently shaped pieces, tetrominos, fall from the top in an apparently random order. The tetromino is a piece formed of four contiguous squares, of which there are seven possible configurations, i.e., there are seven possible geometrical shapes of a tetromino. The piece falls until it reaches the grid floor or another tetromino. The player chooses where the tetromino is placed through rotation and/or horizontal movement. When a row is filled, it is cleared, and all cells above move one row down. The objective is to clear as many rows as possible before the game ends, which is when there is no space remaining at the top of the stacked tetrominos to place a new piece (i.e., the piece crosses the grid top). In its original form, the difficulty level increases as the player clears rows, and the speed of the falling tetromino increases with increased difficulty level (Thiery and Scherrer, 2009), and the tetrominos’ sequence is perfectly random (Burgiel, 1997). Regardless of the players’ skill level, the game will thus invariably end.

Several modifications were made to ensure equal conditions for participants. The duration of all games was set to 4 min before automatically ending, which also closed the gameplay’s interface. If a game ended before 4 min, the game was programmed to restart automatically. The difficulty level displayed originally was hidden from participants to avoid expectancy bias and remove this potential indication of workload. Participants were thus uninformed of the difficulty level. Some versions of Tetris include visual aids, such as displaying gridlines or a “ghost” in the game space. The “ghost” of the tetromino displays a shadow of what the tetromino would look like if it were permanently placed directly below its current position. Our version did not include a “ghost” or gridlines, but it did include a preview window that displayed the next tetromino.

#### 2.1.2 Conditions and tasks

We manipulated workload by means of creating three Tetris gameplays. One ***Easy*** condition and one ***Hard*** condition featured a low and high difficulty levels, respectively, both held constant throughout the 4 min duration. The third condition, ***Ramp***, had increasing difficulty levels throughout its 4-min duration. One practice gameplay was also created. Table 1 provides the difficulty levels. Each participant underwent all three conditions. Participants were sequentially assigned to one of six groups following a 3 × 3 Latin Square Design.

**Table 1:** Tetris parameters for each experimental condition with a base speed set at 0.400 s (see Supplementary material for explanation).

Condition	Start level	End level	Increments	Duration (min)
Practice	1	5	5	4
Easy	1	1	0	4
Hard	12	12	0	4
Ramp	1	15	6	4

The primary task was to perform well in the Tetris gameplay. Participants were additionally exposed to five alarms triggered at pseudorandom intervals (see Supplementary material) during each Tetris gameplay, and their secondary task was to turn the alarm off as quickly as possible.

#### 2.1.3 Experimental procedure

**Figure 1:**
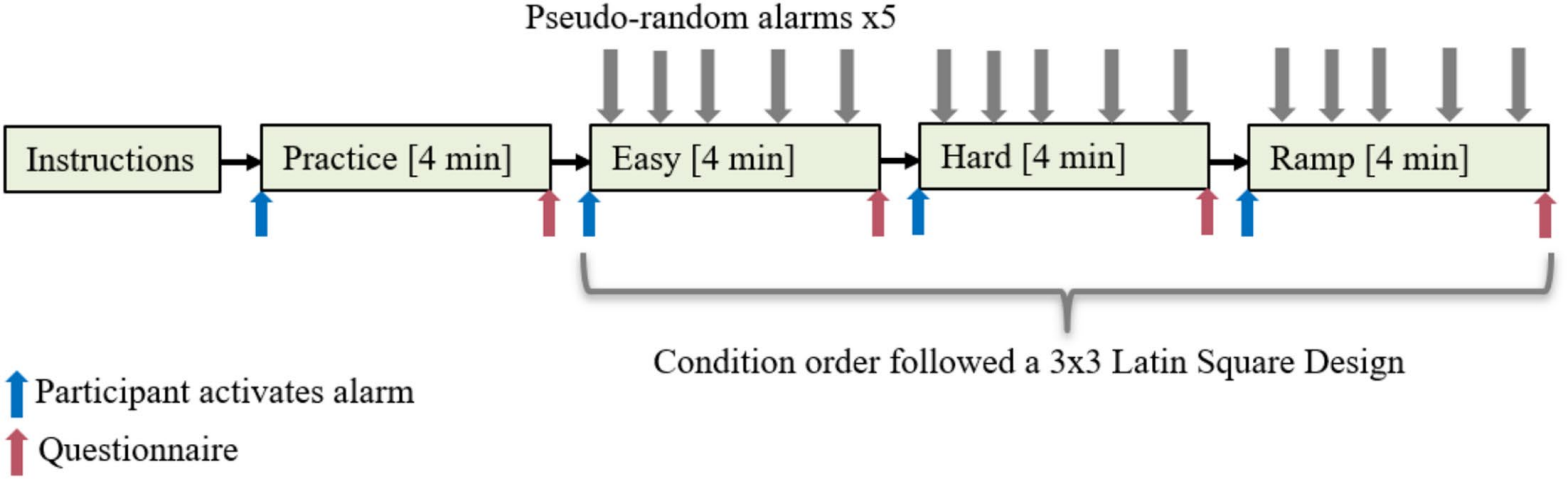
Experiment procedure.

Upon arrival, participants were informed by the experimenter that they would be playing Tetris and reacting to an alarm as quickly as possible. Their task was to get as high a score as possible in all the games they played, while simultaneously turning the alarm off as soon as possible. Their performance would be evaluated both on reaction time and Tetris score. The participant performing the best would be awarded a gift certificate. Participants were fitted with sensors after signing a consent form. Remaining task instructions, i.e., how to play Tetris and use the alarm, were given on screen, ensuring every participant received the same explanation. Before each game, participants were instructed to activate the alarm by flipping a switch once; the same switch would turn the alarm off. A demographic questionnaire was completed after the last condition. Figure 1 provides an overview of the experiment procedure.

### 2.2 Participants

Participants were recruited through posters placed around campus. A large text advertised the possibility to win a 1000 NOK gift certificate at a local mall, accompanied by a smaller text that stated participants were needed for an experiment in which they would play Tetris. At the same time, their brain activity would be measured.

Thirty-two healthy adults participated in this study. Participants were aged above 18 years and understood written and spoken English. Exclusion criteria eliminated individuals with neurological disorders or who were using medication affecting brain function (e.g., stimulants and antidepressants). As the experiment was conducted when COVID-19 infection control measures were in place, participants were required to present a negative test result on a recent coronavirus test and adhere to local infection control measures. The study was approved ethically according to local regulations. Participants provided written informed consent before participating. Caffeine intake was not controlled for. Two participants were excluded from all data analysis due to non-compliance with the experimental procedure, leaving *N* = 30 valid participants. The age of participants ranged from 19 to 42 years (*M* = 26.1, SD = 4.2). Table 2 provides additional demographic information.

**Table 2:** Demographical information (*N* = 30).

Variable	Category	Number of participants
Gender	Female	11
	Male	19
Handedness	Right handed	27
	Left handed	3
Students	Yes	27
	No	3
Have you played Tetris in the past?	Yes	23
	No	7
Do you play Tetris currently?	Weekly	1
	Monthly	1
	Yearly	7
	No	21
Have you played other video games in the past?	Yes	27
	No	3
Do you play video games currently?	Daily	3
	Weekly	4
	Monthly	4
	Yearly	10
	No	9

### 2.3 Hypotheses

fNIRS and EEG data were split into 60-s blocks, yielding four blocks per condition. ECG data were divided into 120-s blocks, yielding two blocks per condition. EDA data were not divided into blocks. Pairwise comparisons between the three conditions were carried out for EDA variables, performance variables, subjective variables, and reaction time because they cannot be split into blocks. For EDA, we expect that SCR relates to task and alarm, and SCL relates to task only—we are therefore interested in the differences in SCL between conditions. We hypothesize that higher levels of SCL are associated with Hard compared to Easy. We expect a difference between Hard and Ramp, but do not have a directional hypothesis *a priori*.

The following hypotheses and contrasts were defined:

- **H1: There is an effect of workload on cognitive load and stress**.
  - Manipulation check: To confirm that conditions Easy and Hard accurately represented low and high workload, respectively, we contrasted Easy and Hard.
    - fNIRS and EEG: As these conditions could be temporally affected, to avoid potential confounds with the temporal effect (H2), we contrasted the first minute of each game. Contrast: Hard1-Easy1.
    - ECG: For the same reason, we contrast the first half of each game. Contrast: Hard1-Easy1.
  - Cognitive load increases linearly with increasing workload, up to a certain threshold (depending both on workload and time), after which mental fatigue occurs. fNIRS and EEG: We run all pairwise contrasts within Ramp. Contrasts: Ramp4-Ramp2, Ramp2-Ramp1, Ramp3-Ramp2, Ramp4-Ramp3, Ramp3-Ramp1, Ramp4-Ramp1.
  - Stress response will be greater with increasing workload. ECG: The second half of Ramp will yield a higher stress response. Contrast: Ramp2-Ramp1.
- **H2: There is a temporal effect of workload on cognitive load and stress, regardless of the actual workload (i.e., difficulty level)**.
- fNIRS and EEG: The first and last minutes of Easy and Hard will have different cognitive loads. Contrasts: Easy4-Easy1, Hard4-Hard1.
- ECG: The first and second halves of Easy and Hard conditions have different stress responses. Contrasts: Easy2-Easy1 and Hard2-Hard1.

### 2.4 Data collection

**Figure 2:**
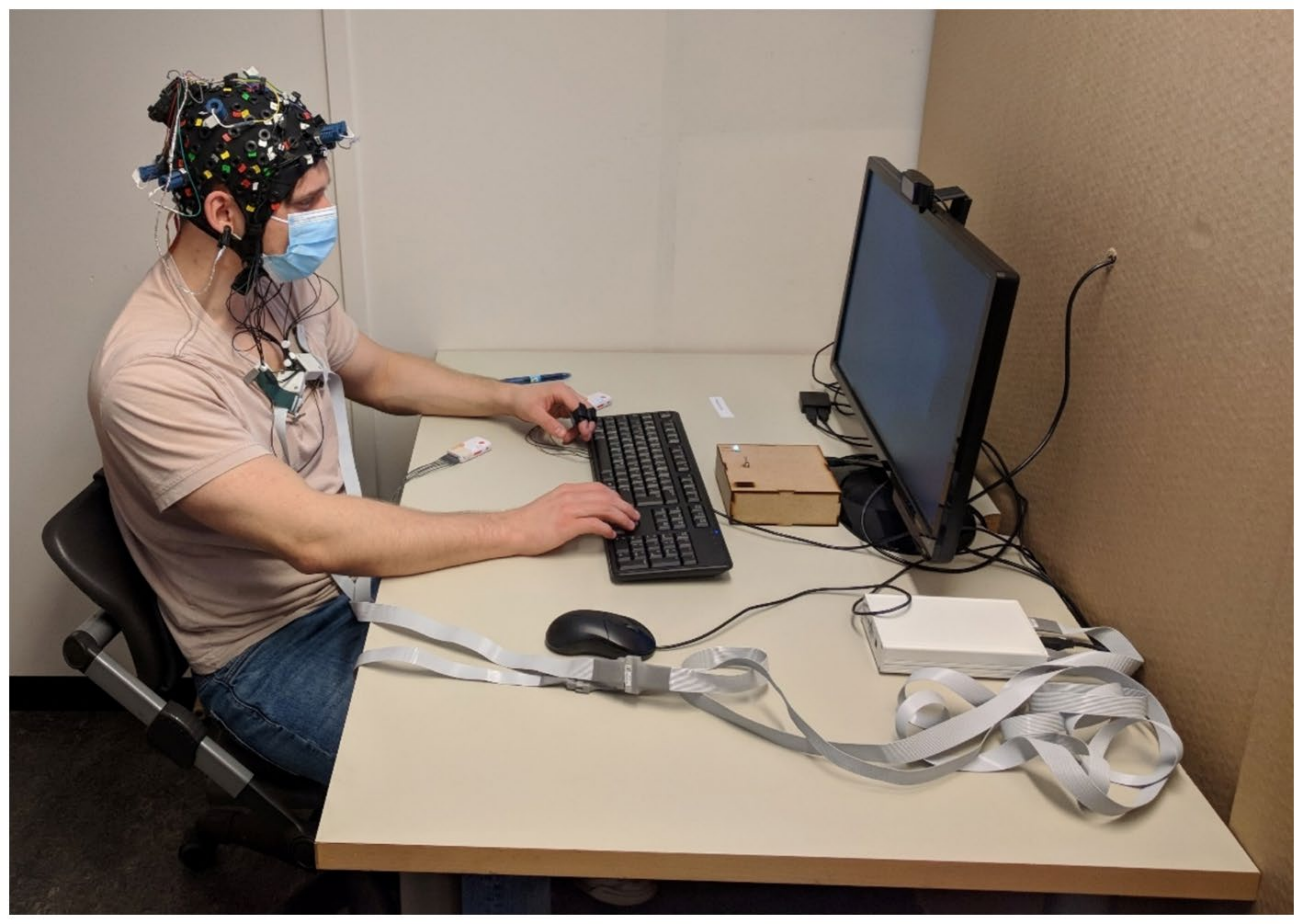
Physical setup.

iMotions version 8.1 (iMotions, Boston, MA, United States) presented stimuli and synchronized neuroimaging, physiology data, and video recordings. An external web camera recorded participants from above; their screen was also recorded. The experiment was run on a Dell Latitude 7,490 laptop (Microsoft Windows 10 Education, Intel(R)Core(TM) i7-8650U CPU@1.90GHz 2.11GHz processor, 32.0GB RAM, 64-bit operating system, ×64-based processor, and a 500GB SSD hard drive). Honeycomb cardboard separated this laptop and the experimenter from the participants. Participants used an external monitor, mouse, and keyboard to interact (Figure 2).

#### 2.4.1 fNIRS data

**Figure 3:**
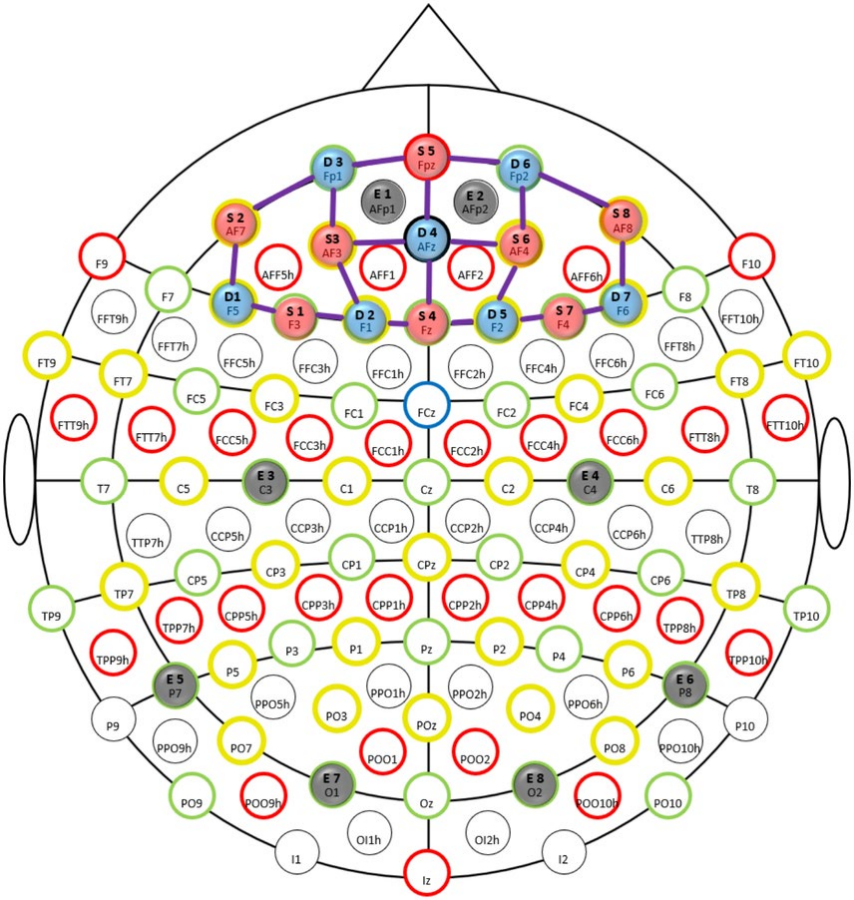
Montage: fNIRS optodes and EEG electrode locations illustrated in the international 10–5 system (Oostenveld and Praamstra, 2001). Sources are indicated in red (8 pieces), detectors in blue (7 pieces), and thick purple lines illustrate channels (20 pieces). Electrodes are indicated in gray (8 pieces).

**Figure 4:**
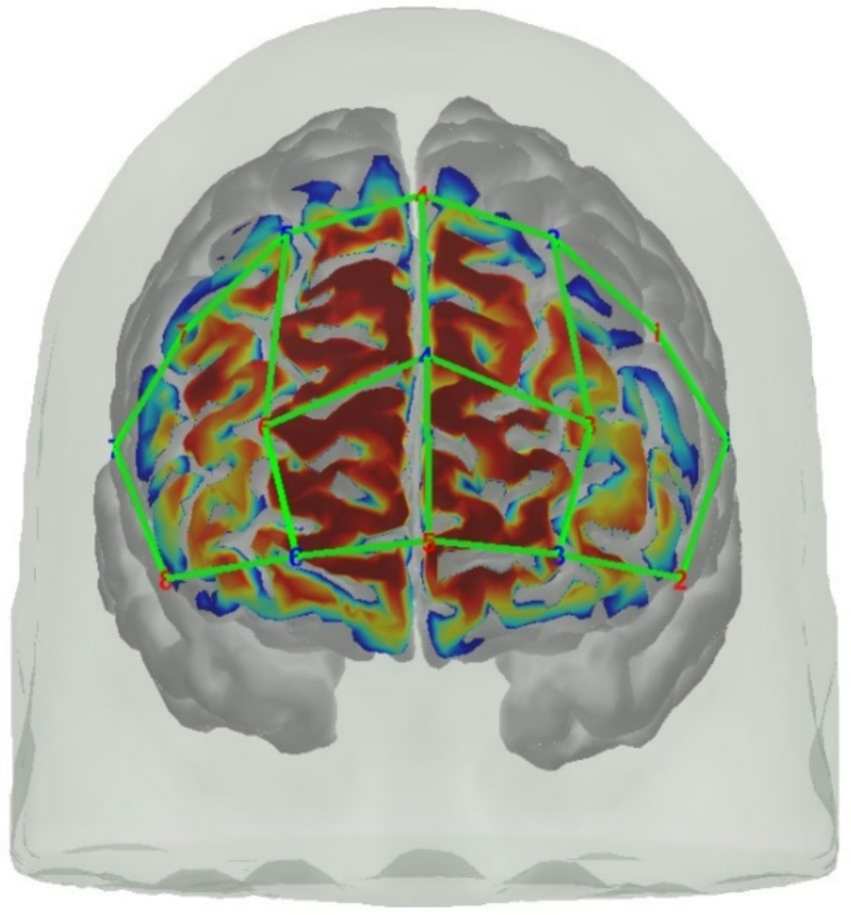
Visualization of the fNIRS montage on a digital brain model and its sensitivity profile generated with AtlasViewer (Aasted et al., 2015).

fNIRS data were sampled at 7.81 Hz with an 8 source/8 detector continuous-wave NIRSport (NIRx Medical Technologies, Berlin, Germany) at two wavelengths (760 and 850 nm) using Nirstar 15.2 Acquisition Software. Optodes were placed on the prefrontal cortex using an EASYCAP AC-128-X1-C-58 (EASYCAP GmbH, Herrsching, Germany) with a 128-channel layout following the 10–5 system (Oostenveld and Praamstra, 2001) (see Figures 3, 4). This montage covers the frontopolar area (PFC), orbitofrontal cortex (OFC), and the dorsolateral prefrontal cortex (dlPFC) (Okamoto et al., 2004; Zimeo Morais et al., 2018). AtlasViewer (Aasted et al., 2015) was used to generate a sensitivity profile (see Figure 4).

Signal quality control was performed, and channels were visually inspected for a visible cardiac oscillation before streaming raw data continuously to iMotions through LabStreamingLayer (n.d.). Optode wires were routed straight down on both sides of participants’ heads before being routed backward to reduce their potential noise in EEG signals.

#### 2.4.2 EEG data

EEG data were sampled at 250 Hz with an OpenBCI Cyton biosensing board (OpenBCI Inc.) using eight spring-loaded dry comb electrodes (provided as part of an Ultracortex “Mark IV” EEG Headset), attached to the EASYCAP using an adapter (Erichsen et al., 2020). The board was wirelessly connected to the laptop via a USB dongle. Electrodes were distributed over the scalp (see Figure 3).

A wooden applicator was used to part the participants’ hair at each electrode location before inserting the spring-loaded electrode. Signal quality control was performed in the OpenBCI GUI, visually inspecting for derailed electrodes and noise, in which case the electrode in question would be refitted. In some instances, salted water was used to help hair stay parted, and thus the electrode retained skin contact and improved electrical connectivity to the scalp. A Python script used the BrainFlow Python library to connect to the Cyton board and pylsl to stream data continuously to iMotions through LabStreamingLayer (n.d.).

#### 2.4.3 ECG data

ECG data were sampled at 512 Hz with a Shimmer ECG sensor (Shimmersense, 2017) using a five-lead configuration with gel pads fastened on participants’ chests. The unipolar lead was mounted at position V_5_ as this allows for the highest quality R-wave capture (Shimmer3 User Guide). Data was streamed directly to iMotions via a Bluetooth connection to the laptop, and the signal was visually inspected for noise in iMotions before recording data.

#### 2.4.4 EDA data

EDA data were sampled at 128 Hz with a Shimmer Galvanic Skin Response sensor (Shimmersense, 2017) which had a two-lead configuration connected to the underside of the medial phalanx on the index and middle finger of the participants’ left hand. Data was streamed directly to iMotions via a Bluetooth connection to the laptop, and the signal was visually inspected for noise in iMotions before data recording commenced.

#### 2.4.5 Reaction time

A secondary ART was implemented with a custom-built Arduino device (Erichsen, 2020). Participants were exposed to five alarms during each Tetris gameplay and assigned to turn the alarm off as quickly as possible, using a panel mount toggle switch mounted on the Arduino device. The device measured reaction time, that is, the time it took participants to turn the alarm off. The alarms were pseudo-randomized, which means that while alarm timing was generated randomly, these timings were used for all conditions and participants. The alarm timings were: 8,000 ms (8 s), 105,000 ms (1 min 45 s), 131,000 ms (2 min 11 s), 169,000 ms (s), 235,000 ms (3 min 55 s). The alarm would stop automatically after 3,000 ms (3 s).

#### 2.4.6 Performance

The number of games and scores for each game were generated by the Tetris game and saved as separate text files.

#### 2.4.7 Subjective data collection

Several subjective variables were measured after each game. Arousal and Valence from the Circumplex Model of Affect (Russell, 1980) was rated using the Affect Grid (Russel et al., 1989), on a scale from 1 (low) to 9 (high). Workload was assessed using the Overall Workload (Vidulich and Tsang, 1987) and NASA Task Load Index (TLX) (Hart and Staveland, 1988) dimensions^1^: Physical Demand, Temporal Demand, Performance, Effort, and Frustration. Participants reported Level of Stress and Enjoyment on a scale from 1 (low) to 7 (high), and Workload Acceptability on a scale from 1 (low) to 7 (high) (unacceptable, this was very hard-highly acceptable, this was easy).

### 2.5 Data analysis

All data were exported as one synchronized “.csv-file” per participant from iMotions. To obtain spatial information about optodes’ positions, a sample recording in NIRStar was made separately. FNIRS data was analyzed, and EEG data preprocessed in NIRS Brain AnalyzIR Toolbox (nirs-toolbox) (Santosa et al., 2018) in MATLAB R2021b (The MathWorks, Inc., Natick, MA, United States). Both fNIRS and EEG timeseries were trimmed to 60 s/120 s before/after the first/last stimuli. EEG frequency bands, EDA variables, performance, arousal, and valence were analyzed with R version 4.2.0 (2022-04-22 ucrt) (R Core Team, 2022) in RStudio 2022.02.2 (RStudio Team, 2022). ECG variables, remaining subjective variables, and reaction time were analyzed with Statistical Package for the Social Sciences (SPSS) version 28.0 (IBM Corporation, 2021). A significance level of *p* < 0.05 was used unless otherwise noted. Table 3 presents an overview of all variables included in the analysis.

**Table 3:** Variables.

Measurement modality	Variable	Description
fNIRS	HbO	Oxygenated hemoglobin
	HbR	Deoxygenated hemoglobin
EEG	Delta power	EEG spectral power band 1–4 Hz
	Theta power	EEG spectral power band 4–8 Hz
	Alpha power	EEG spectral power band 8–12.5 Hz
	Beta power	EEG spectral power band 12.5–30 Hz
ECG	MeanRR [ms]	Mean RR interval
	SDNN [ms]	Standard deviation of RR intervals
	MeanHR [bpm]	Mean heart rate
	SDHR [bpm]	Standard deviation of heart rate
	RMSSD [ms]	Root mean square of successive differences
	pNN20	pNN20 (%) Percentage of successive intervals that differ more than 20 ms. Proposed to assess PNS (Mietus et al., 2002).
	LFpeak [Hz]	Peak frequency for LF band
	HFpeak [Hz]	Peak frequency for HF band
	LFpow [ms^2^]	Absolute LF power
	HFpow [ms^2^]	Absolute HF power
	LFpow [log]:	Log LF power
	HFpow [log]:	Log HF power
	LFpow [%]	Relative LF power
	HFpow [%]	Relative HF power
	LFpow [n.u.]	Normalized LF power
	HFpow [n.u.]	Normalized HF power
	LF_HF_ratio [n.u.]	Ratio between LF and HF powers
EDA	nSCR [n.u.]	Number of significant (above-threshold) SCRs within the response window (wrw)
	Latency [s]	Response latency of first significant SCR wrw
	AmpSum [μS]	Sum of SCR-amplitudes of significant SCRs wrw
	SCR [μS]	Average phasic driver wrw.
	ISCR [μS*s]	Area (i.e., time integral) of phasic driver wrw.
	PhasicMax [μS]	Maximum value of phasic activity wrw
	Tonic [μS]	Mean tonic activity wrw
	Mean [μS]	Mean SC value wrw
	MaxDeflection	Maximum positive deflection wrw
Reaction time	Reaction time [ms]	The time from the start of the alarm sound until the participant switched the alarm off. 5 times per condition.
Questionnaire (subjective variables)	Arousal	Describe how you feel right now by using the Affect Grid. Arousal [1–9 point scale, 9 = high arousal, 1 = low arousal].
	Valence	Valence [1–9 point scale, 1 = unpleasant, 9 = pleasant].
	Level of stress	How would you rate your level of stress during the preceding Tetris game? Rate level of stress on a scale from 1 (low) to 7 (high).
	Overall workload	Overall workload was rated on a scale from 1 (low) to 7 (high).
	TLX: Physical demand	How much physical activity was required? (e.g., pushing, pulling, turning, controlling, activating, etc.) Rate physical demand on a scale from 1 (low) to 7 (high).
	TLX: Temporal demand	How much time pressure did you feel due to the rate or pace at which the tasks or task elements occurred? Rate temporal demand on a scale from 1 (low) to 7 (high).
	TLX: Performance	How successful do you think you were in accomplishing the goals of the tasks? Rate performance on a scale from 1 (poor) to 7 (good).
	TLX: Effort	How hard did you have to work (mentally and physically) to accomplish your level of performance? Rate effort on a scale from 1 (low) to 7 (high).
	TLX: Frustration	How insecure, discouraged, irritated, stressed, and annoyed vs. secure, gratified, content, relaxed, and complacent did you feel during the task? Rate frustration on a scale from 1 (low) to 7 (high).
	Enjoyment	Enjoyment was rated on a scale from 1 (low) to 7 (high).
	Workload acceptability	How acceptable was the workload? Rate workload acceptability on a scale from 1 (unacceptable—this was very hard) to 7 (highly acceptable—this was easy).
Performance	Number of games played	The total number of Tetris games played by participants in the condition.
	Average score per game	The average score per Tetris game played in the condition.
	Total score	The sum of all scores from all Tetris games played in one condition.

#### 2.5.1 fNIRS analysis

The NIRStar sample recording was combined with raw fNIRS data to obtain a data structure with correct spatial information, before visual inspection to ensure no missing data. To assess signal quality, we calculated the Coefficient of Variance (CV) of the raw data, discarding channels with CV > 0.1. 97% of the data had CV < 0.1 and was retained for further analysis. Raw light intensities were converted to optical density before converting to HbO and HbR through the modified Beer–Lambert Law with a partial pathlength factor of 0.1 and extinction coefficient from Jacques (2013). Afterward, participant-level statistics were calculated using a general linear model (GLM) with a canonical hemodynamic response function that employed an autoregressive, iteratively reweighted least-squares model (AR-IRLS). This approach uses an autoregressive (AR) prewhitening filter to alleviate serially correlated errors resulting from physiological noise and/or motion artifacts. This AR-whitened model is then solved using robust weighted regression, which iteratively down-weight outliers to address heavy-tailed noise from motion artifacts (Barker et al., 2013, 2016). Using this model, the regression coefficients (*β*) and their error-covariance are estimated, which are used to define statistical tests between task conditions or baseline. Leverage for a group model was calculated across participants, conditions, and channels, but no participant contributed significant leverage, and thus all participants were retained. For group-level statistics, we ran a robust mixed-effects model that included condition as a main effect and participant as a random effect (Santosa et al., 2018). These results were used for group-level contrasts (*t*-tests) between conditions. The Benjamini–Hochberg procedure was used to control false-discovery rate (FDR) (Benjamini and Hochberg, 1995). The corrected *p*-value is denoted as *q*, and *q* < 0.05 is used as a significance threshold. Results are reported as maps depicting group-level activation of HbO and HbR as per best practice (Yücel et al., 2021).

#### 2.5.2 EEG analysis

Raw EEG data were visually inspected to assess data quality. Five participants were discarded due to flatlined data and missing conditions, leaving 25 participants for the subsequent analysis. Preprocessing included bandpass filtering (1–48 Hz) to attenuate line noise and remove low amplitude. As less preprocessing yields higher statistical sensitivity (Delorme, 2023) no further preprocessing was undertaken before frequency analysis. Frequency bands were defined as follows: Delta: 1–4 Hz; Theta: 4–8 Hz; Alpha 8–12.5 Hz; and Beta: 12.5–30 Hz (NiederMeyer, 2011). Frequencies were computed using a continuous wavelet transform, transforming the timeseries to the frequency domain. Data was down-sampled to 4 Hz before running a block analysis model on the frequency domain results with the AR-IRLS algorithm and a canonical basis. This yielded one beta value representing the frequency power per frequency band and electrode for each condition. This beta power was carried forward in group-level analysis, undertaken in R. The results were fed into a mixed-effects model [*lme4::lmer* (Bates et al., 2015)] including the main effect of condition and participant as a random effect. Individual models were set up for each frequency and electrode. The individual mixed-effects models were used to estimate marginal means (*emmeans::emmeans*) and test contrasts (*emmeans::contrast*) (Lenth, 2022). The Benjamini–Hochberg procedure (Benjamini and Hochberg, 1995) controlled FDR. Frequency bands are presented as estimated differences (standard error).

#### 2.5.3 ECG analysis

ECG data were visually inspected to assess data loss. Participants with full or partial data loss were excluded, leaving N = 25 for subsequent analysis. ECG data were preprocessed in Kubios HRV Premium (Tarvainen et al., 2014), using the LL-RA ECG lead. Kubios uses a QRS detection algorithm based on the Pan–Tompkins algorithm (Pan and Tompkins, 1985) for R-peak detection. Preprocessing included bandpass filtering (to reduce baseline wander, power line noise, and other noise components), squaring data samples to highlight peaks, up-sampling through interpolation to improve time resolution of R-peak detection, and artifact correction (Tarvainen et al., 2014, 2021). The data and detected R-peaks were visually inspected in Kubios. Artifacts were corrected or marked as noise. Thereafter, HRV variables in the time and frequency domains were obtained. For the frequency domain, we adjusted the VLF band’s lower limit to 0.0033 (it was 0). The LF and HF bands were 0.04–0.15 and 0.15–0.40 Hz, respectively. We used an AR model with a default model order of 16, because AR models exhibit increased robustness and accuracy for shorter recordings (Malliani et al., 1991; Montano et al., 2009). Consistent with general guidelines, the frequency-domain variables are reported in absolute and normalized forms to present a complete picture of the power distribution (Malik et al., 1996). The standard pNN value of 50 ms was changed to 20 ms because it consistently enhanced discrimination ability (Mietus et al., 2002).

Recording length restricts frequency-domain measurements, specifically the HRV frequency-band measurements (Shaffer and Ginsberg, 2017). Minimum recommended periods include: VLF (5 min), LF (2 min), and HF (1 min) (Shaffer and Ginsberg, 2017). As we wanted to obtain LF measures, we sliced each condition into two blocks of 2 min each.^2^ As such, we could not slice one condition into four 1-min blocks as we did for the fNIRS/EEG analysis.

We ran a repeated measures ANOVA version of the GLM with custom contrasts. GLM assumptions were assessed by visually examining histograms, boxplots, and Q–Q plots. Some variables had outliers and deviated from sphericity; hence, we routinely apply and interpret the Greenhouse–Geisser correction. For corroboration, we ran Friedman test and robust method with a bootstrap of 599 samples and 20% trim (via *WRS2::rmanovab* in R, see 2.5.4 for details). As the Friedman test and robust approach do not allow custom contrasts we report the custom contrasts from the GLM. The contrast involving Ramp1 and Ramp2 is independent from the remaining contracts; thus, the level of significance was set at *p* < 0.05. The contrasts involving Hard and Easy are non-orthogonal. To control the familywise error rate for these contrasts, we set and interpret a Bonferroni corrected level of significance *p* = (0.005/3) = 0.0167. MD denotes the contrast estimate of the difference in mean. Partial η_p_^2^ estimates the effect size for the contrasts. ECG variables are reported as mean difference and 95% confidence interval (CI).

#### 2.5.4 EDA analysis

EDA data were visually inspected for missing data, artifacts, and lack of EDA response. Two participants without an EDA response were discarded from subsequent analysis. EDA data were processed in Ledalab (Benedek and Kaernbach, 2010). We preprocessed data manually in cases with missing data or many artifacts. Times with missing data were removed, significant artifacts were marked manually and corrected with spline interpolation, and minor artifacts were smoothed. The remaining data were batch processed. The data were downsampled to 16 Hz. The data was analyzed with Continuous Decomposition Analysis (CDA) as it is more robust to motion artifacts, and estimates tonic activity better than standard through-to-peak and deconvolution methods (Benedek and Kaernbach, 2010). We chose CDA because it was essential to attain an accurate estimate of tonic activity related to the task.

GLM assumptions were assessed via scatterplots, histograms, and Q–Q plots. In cases without violations of assumptions (nSCR), we used a repeated measures model [*afex::aov_4* (Singmann et al., 2022]. For normally distributed heteroscedastic variables (Latency, SCR, PhasicMax, Mean, and MaxDeflection), we used a robust method that uses a 20% trimmed mean (*WRS2::rmanova:* a heteroscedastic one-way repeated measures ANOVA for trimmed means) (Mair and Wilcox, 2020). This implementation simulates a critical *p*-value before computing the p-value accordingly. The p-value (p) should thus be compared to a critical *p*-value (*p*-critical). For non-normally distributed heteroscedastic variables (Tonic, AmpSum, and ISCR), we used a robust method with a bootstrap of 599 samples (*WRS2::rmanovab*: a bootstrap version of the heteroscedastic one-way repeated measures ANOVA) (Mair and Wilcox, 2020). This implementation simulates a critical value (*t*-critical), to which the test statistic (*t*) should be compared.

#### 2.5.5 Performance

The number of games and scores for each condition were used to create three performance variables: number of games, total score (summarizing all scores for all games), and average score per game. A high total score indicates high performance and average score per game, and a low number of games played (because fewer games means the participant had fewer games ending due to not being able to clear enough rows (i.e., “died” less)). The assumptions of the GLM, assessed via scatterplots, histograms, and Q–Q plots, were violated. Number of games was not normally distributed and heteroscedastic, average, and total scores also had outliers. We therefore used a robust method (*WRS2::rmanova*) and associated *post hoc* tests (*WRS2::rmmcp*) (Mair and Wilcox, 2020). Effect sizes (Cohen’s d) were estimated from Bonferroni-corrected contrasts obtained from a regular repeated measures model [*afex::aov_4* (Singmann et al., 2022)] that used untrimmed means [*emmeans::emmeans* (Lenth, 2022) and *effectsize::t_to_d* (Ben-Shachar et al., 2020)]. For the robust methods, *Ψ* indicates the estimated mean difference between conditions. Performance variables are reported as mean (standard deviation).

#### 2.5.6 Subjective variables

Arousal and valence were heteroscedastic and deviated from normality, thereby violating the assumptions of the GLM, as assessed via scatterplots, histograms, and Q–Q plots. Thus, we used a robust method with a bootstrap of 599 samples (*WRS2:*:*rmanovab*) and associated post hoc tests (*WRS2::pairdepb*) (Mair and Wilcox, 2020). Effect sizes (Cohen’s d) were estimated from Bonferroni-corrected contrasts obtained from a regular repeated measures model [*afex::aov_4* (Singmann et al., 2022)] that used untrimmed means [*emmeans::emmeans* (Lenth, 2022) and *effectsize::t_to_d* (Ben-Shachar et al., 2020)].

For the remaining subjective (self-report) variables, differences between conditions were assessed using Friedman’s test. Pairwise comparisons of conditions were carried out using Wilcoxon’s test with a Bonferroni correction for multiple comparisons. Pearson’s correlation coefficient, *r*, estimated effect size (Field, 2018; Rosenthal, 1991) for each pairwise comparison. For corroboration, we ran a 1-way repeated measures ANOVA. Subjective variables are reported as mean (standard deviation).

#### 2.5.7 Reaction time

Reaction times were approximately normally distributed as assessed by histograms and normal Q–Q plots, but had multiple outliers. We ran a factorial repeated-measures ANOVA model with game difficulty level and alarm number as independent variables. All effects in the factorial model deviated from sphericity. Thus, we adjust the degrees of freedom using the Greenhouse–Geisser estimate of departure from sphericity (*ε*). We ran a mixed-effects linear model for corroboration that included difficulty levels, alarm, and alarm–difficulty interaction as fixed effects and a random intercept.

## 3 Results

### 3.1 fNIRS

#### 3.1.1 Assessing H1

##### 3.1.1.1 Manipulation check

**Figure 5:**
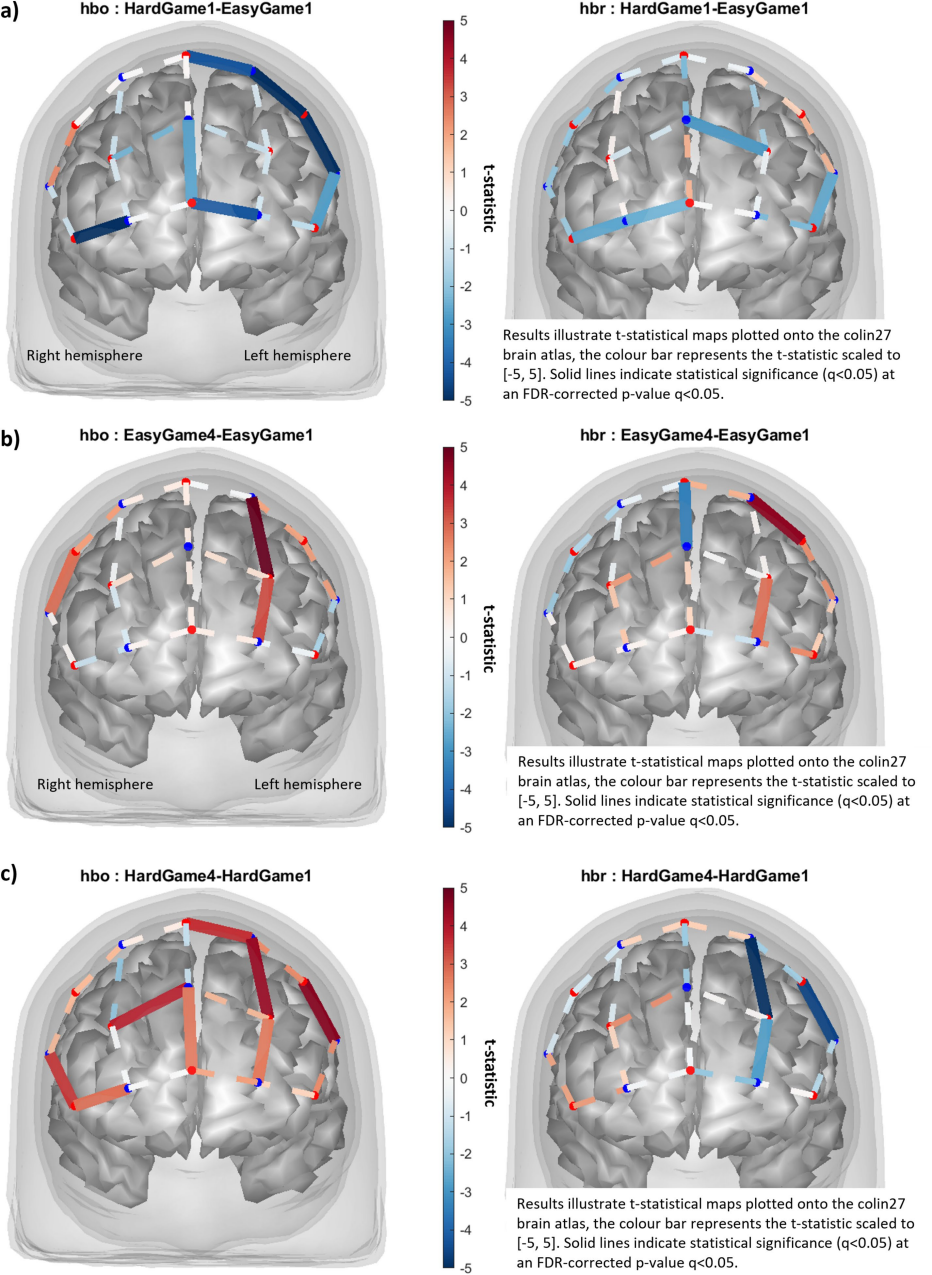
**(a)** Contrast comparing the first minutes of Hard and Easy, **(b)** contrast comparing the last and the first minutes of Easy, **(c)** contrast comparing the last and the first minutes of Hard.

The first minutes of Easy and Hard were contrasted to ensure that the conditions accurately represented low and high difficulty levels, i.e., that Hard was more cognitively demanding than Easy. Seven channels had significant HbO decrease (left dlPFC, FPC) and four channels had significant HbR decrease (FPC, left dlPFC) in Hard1 compared to Easy1 (Figure 5a). The HbR data suggest cognitive load was higher in Hard1, while the HbO data suggest higher cognitive load in Easy1. See Supplementary material for statistics tables.

##### 3.1.1.2 Contrasts within ramp

To further understand the effects of increasing difficulty levels on cognitive load, we contrasted all minutes within Ramp, pairwise. See Supplementary material for figures and statistics. For Ramp2-Ramp1, two channels had a significant HbR increase, suggesting higher cognitive activation in Ramp1 than Ramp2. For Ramp3-Ramp2, one channel had a significant HbO decrease, one significant HbR decrease, and one significant HbR increase. HbO data suggests higher activation in Ramp2 than Ramp3, while HbR is mixed. For Ramp4-Ramp3, one channel had a significant HbO decrease and one significant HbR increase, suggesting higher activation in Ramp3 than Ramp4. For Ramp4-Ramp2, six channels had significant HbO decrease, and four channels had significant HbR increase. The higher activation in Ramp2 compared to Ramp4 suggests higher cognitive activation in Ramp2 compared to Ramp4. For Ramp3-Ramp1, two channels had significant HbO decrease, four channels had significant HbR decrease, and one channel had significant HbR increase. These HbO data suggest higher activation in Ramp1 than Ramp3, while HbR data are mixed. For Ramp4-Ramp1, seven channels had significant HbO decrease, three channels had significant HbR decrease, and five channels had significant HbR increase. This suggests higher cognitive activation in Ramp1 compared to Ramp4. Overall, these results suggest cognitive activation is highest at the start of the gameplay, and as the difficulty level increases, cognitive activation reduces.

#### 3.1.2 Assessing H2

##### 3.1.2.1 Contrasting first and fourth minutes in constant load conditions

To investigate whether there was a temporal effect of workload (H2), irrespective of difficulty levels, we contrasted the fourth to the first minutes for both Easy and Hard. For Easy4-Easy1 (Figure 5b) three channels had significant HbO increase (right dlPFC, midt-left OFC/dlPFC), one significant HbR decrease (midline), and two significant HbR increases (midt-left OFC/dlPFC). The higher activation in Easy4 suggests it is more cognitively demanding than Easy1, supporting H2. For Hard4-Hard1 (Figure 5c), eight channels had significant HbO increase (across the PFC), and three channels had significant HbR decrease (left PFC). The higher activation in Hard4 suggests it is more cognitively demanding than Hard1, supporting H2. See Supplementary material for statistics tables.

### 3.2 EEG

#### 3.2.1 Assessing H1

##### 3.2.1.1 Manipulation check

The first minutes of Easy and Hard were contrasted to ensure that the conditions accurately represented low and high difficulty levels, that is, that Hard was more cognitively demanding than Easy. For Theta power, there were significant differences for channel O1 [***Δ*** = 3.67 (1.46), *t* = 2.518, *p* = 0.0124] and O2 [**Δ** = 6.01 (2.1), *t* = 2.859, *p* = 0.0046], that is, increased Theta power in Hard1 compared to Easy1. The occipital Theta power increase indicates that Hard1 was more cognitively demanding than Easy1. For Alpha power, one significant channel, O2 [**Δ** = 3.8 (1.9), *t* = 1.998, *p* = 0.0468] indicated an increased Alpha power in Hard1 compared to Easy1. The occipital Alpha power increase further suggests mental fatigue occurred in Hard1 compared to Easy1. For Beta power, there were significant differences for channel P8 [**Δ** = 1.35 (0.654), *t* = 2.067, *p* = 0.0397] and O2 [**Δ** = 1.66 (0.604), *t* = 2.748, *p* = 0.0064], indicating increased Beta power in Hard1 compared to Easy1. The increase in Beta power in the right parietal and occipital regions could indicate higher cognitive load or mental fatigue, depending on to which literature basis it is compared. The remaining channels and frequency bands were non-significant (see Supplementary material).

##### 3.2.1.2 Contrasts within ramp

All frequency bands and channels were non-significant (see Supplementary material).

#### 3.2.2 Assessing H2

##### 3.2.2.1 Contrasting first and fourth minutes in constant load conditions

For Delta power, there was a significant difference between Easy4 and Easy1 for channel P8 [**Δ** = 15.15 (6.12), *t* = 2.476, *p* = 0.0278], indicating higher Delta power in Easy4 compared to Easy1. The right parietal Delta power increase suggests the presence of mental fatigue in Easy4 compared to Easy1. All other frequency bands and channels were non-significant (see Supplementary material).

### 3.3 ECG

#### 3.3.1 Assessing H1

None of the time-domain variables yielded significant differences for any contrasts (see Supplementary material for details).

For HFpeak [Hz], the custom contrasts revealed a significant difference between Ramp2 and Ramp1 (*MD =* −0.048, 95% CI: [−0.088, −0.007], *p =* 0.024, η^2^ = 0.194). The Hard1-Easy1 contrast was non-significant (*MD =* −0.032, 95% CI: [−0.065, 2.22e-04], *p =* 0.051, η^2^ = 0.149).

For HFpow [log], the custom contrasts revealed significant differences between Ramp2 and Ramp1 (*MD =* −0.27, 95% CI: [−0.51, −0.02], *p =* 0.033, η^2^ = 0.176). The Hard1-Easy1 contrast was non-significant (*MD =* −0.09, 95% CI: [−0.28, 0.09], *p =* 0.311, η^2^ = 0.043).

None of the remaining frequency-domain variables yielded significant differences between conditions (see Supplementary material), indicating no difference in participants’ physiological stress response between conditions.

HFpeak [Hz] and HFpow [log] were significantly lower in Ramp2 compared to Ramp1. As HF is modulated by PNS activity, a HF decrease represents lower PNS activity, accompanied by increased SNS activity and stress response. This result suggests that Ramp2 elicited a higher stress response than Ramp, partly supporting H1. There is an effect of difficulty level on stress response, as measured by HFpow [log] and HFpeak. Still, this effect emerges only when the difficulty level incrementally increases over time (i.e., in Ramp). When the difficulty level is constant, the results suggest that high and low difficulty levels cannot be significantly discriminated with HRV.

#### 3.3.2 Assessing H2

None of the time-domain variables or frequency-domain variables yielded significant differences for contrasts assessing H2 (see Supplementary material for details).

### 3.4 Performance

#### 3.4.1 Number of games

**Figure 6:**
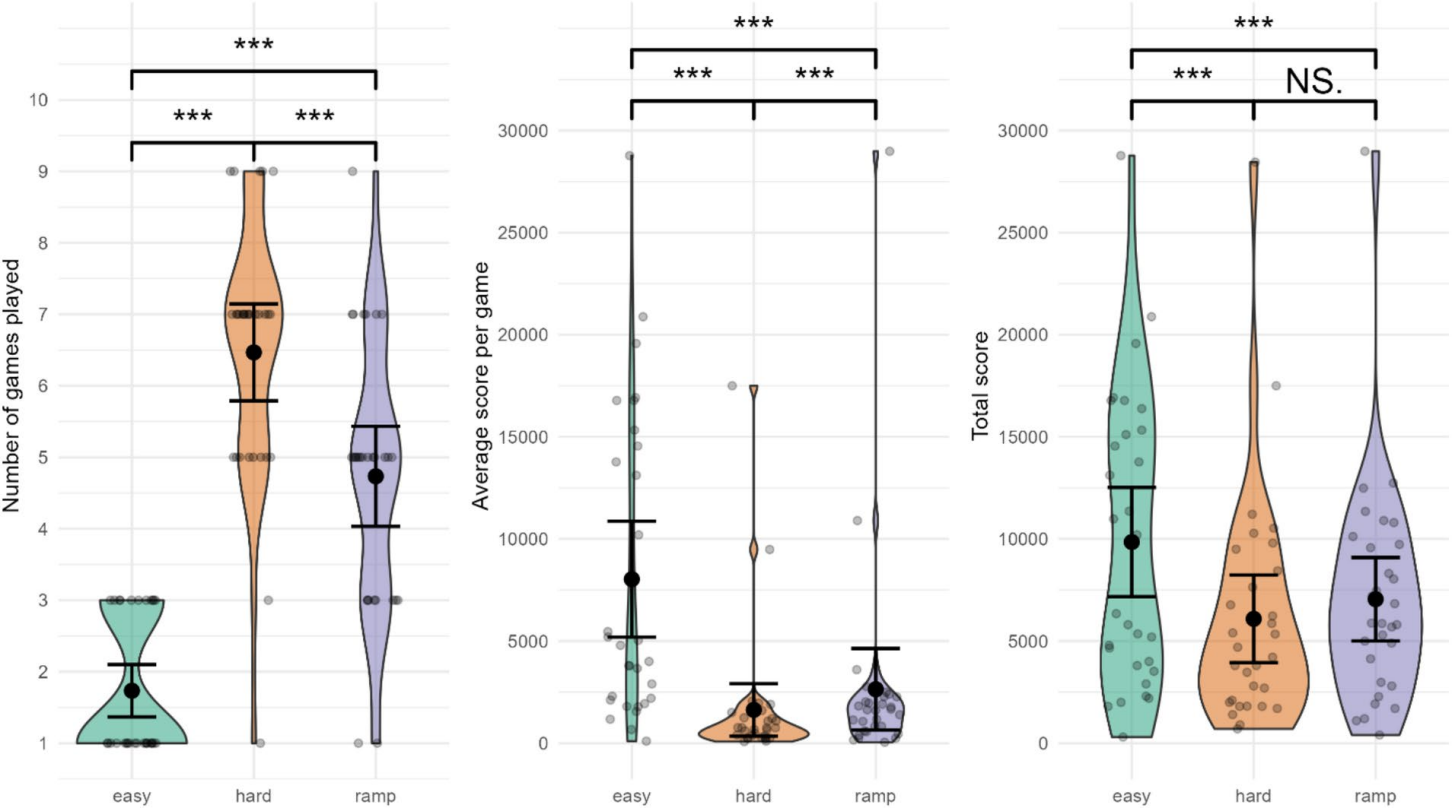
Performance in Tetris gameplay. ^***^Significant difference. NS., not significant. Error bars represent 95% CI assuming normality.

The number of games played was lowest in Easy, *M =* 1.73 (0.98) (indicative of greater performance), followed by Ramp, *M =* 4.73 (1.87) and Hard, *M =* 6.47 (1.81) (see Figure 6). Number of games differed significantly between conditions, F_t_ (1.69, 28.66) = 62.0769, *p =* 0. Pairwise comparisons indicated substantially lower number of games played during Easy compared to Hard (*Ψ* = −4.89, 95% CI: [−5.71, −4.07], *p =* 0e+00 *< p*-critical = 0.0169, *d* = −5.95), and in Easy compared to Ramp (Ψ = −3.00, 95% CI: [−3.82, −2.18], *p =* 0e+00 *< p*-critical = 0.0250, *d* = −3.13). Significantly more games were played in Hard compared to Ramp (Ψ = 1.67, 95% CI: [0.91, 2.42], *p =* 2e-05 *< p*-critical = 0.05, *d* = 2.27).

#### 3.4.2 Average score per game

The average score per game was the greatest in Easy, *M* = 8,031 (7,599), followed by Ramp, *M =* 2,640 (5,340). Hard had the lowest average score per game, *M* = 1,629 (3,439). Average score per game differed significantly between conditions, F_t_ (1.02, 17.26) = 9.7908, *p =* 0.00587. Pairwise comparisons indicated substantially higher average score in Easy compared to Hard (Ψ = 4,954, 95% CI: [712, 9,197], *p* = 0.00649 *< p*-critical = 0.0250, *d* = 1.96), and compared to Ramp (Ψ = 3,793, 95% CI: [−117, 7,704], *p* = 0.01964 *< p*-critical = 0.0500, *d* = 1.76). Hard had a significantly lower average score than Ramp (Ψ = 467, 95% CI: [69, 865], *p =* 0.00629 *< p*-critical = 0.0169, *d* = 0.54).

#### 3.4.3 Total score

The total score was greatest in Easy, *M =* 9,849 (7,163), followed by Ramp, *M =* 6,088 (5,750). Hard had the lowest total score, *M =* 7,049 (5,473). Total score was significantly different between conditions, F_t_(1.42, 24.1) = 7.81, *p =* 0.00527. Pairwise comparisons indicated that the total score was significantly greater in Easy compared to Hard (Ψ = 3,455, 95% CI: [435, 6,475], *p =* 0.00744 *< p*-critical = 0.0169, *d* = 1.55), and Ramp (Ψ = 2,648, 95% CI: [101, 5,195], *p =* 0.01338 *< p*-critical = 0.0250, *d* = 1.27). Total score was not significantly lower in Hard compared to Ramp (Ψ = −828, 95% CI: [−2,173, 518], *p =* 0.12085 *> p*-critical = 0.05, d = 0.71).

### 3.5 Subjective variables

#### 3.5.1 Arousal

**Figure 7:**
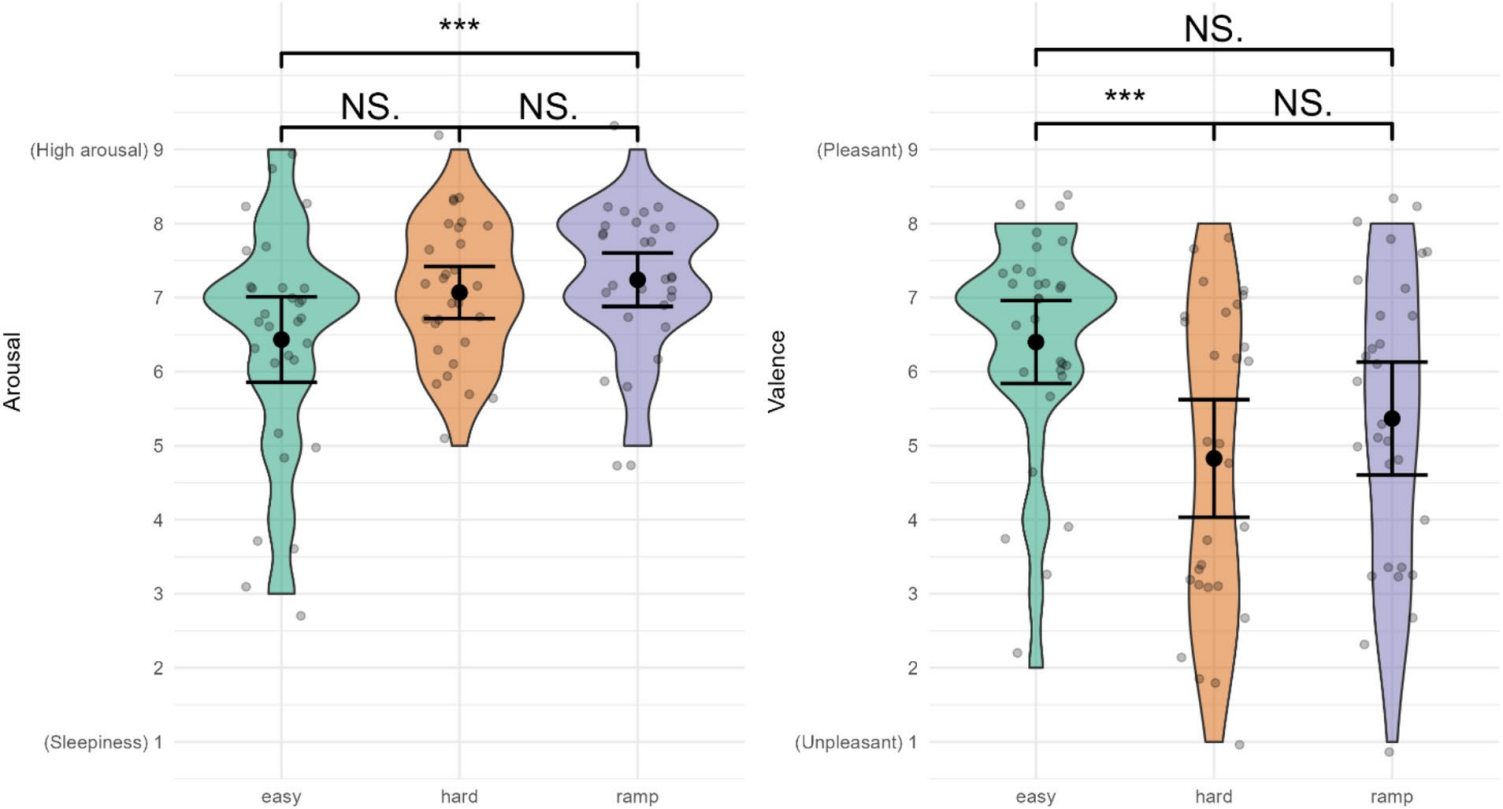
Affective state. ^***^Indicates significant differences, NS., not significant. Error bars represent 95% CI assuming normality.

Arousal (Figure 7) was highest in Ramp, *M =* 7.24 (0.95), followed by Hard, *M =* 7.07 (0.92), with the lowest arousal in Easy, *M =* 6.43 (1.57). There were significant differences between conditions for arousal (*t* = 3.739 > *t*-critical = 3.592). Pairwise comparisons indicated a significantly higher arousal in Ramp compared to Easy (Ψ = 0.78, 95% CI: [0.04, 1.51], *t* = 2.698 > *t*-critical = 2.556, *d* = 1.14). There were no significant differences between Easy and Hard (Ψ = −0.44, 95% CI: [−1.33, 0.44], *t* = −1.279 < *t*-critical = 2.556, *d* = −0.76), or between Hard and Ramp (Ψ = −0.33, 95% CI: [−0.85, 0.19], *t* = −1.638 < *t*-critical = 2.556, *d* = 0.40).

#### 3.5.2 Valence

Valence (Figure 7) was rated most pleasant in Easy, *M =* 6.40 (1.50), followed by Ramp, *M =* 5.37 (2.04). Hard was rated most unpleasant, *M =* 4.83 (2.09). Valence was significantly different between conditions (*t* = 6.160 > *t*-critical = 3.169). Pairwise comparisons indicated that Easy was significantly more pleasant than Hard (Ψ = 1.79, 95% CI: [0.45, 3.12], *t* = 3.423 > *t*-critical = 2.554, *d* = 1.54). There was no significant difference between Easy and Ramp (Ψ = 1.11, 95% CI: [−0.21, 2.42], *t* = 2.139 < *t*-critical = 2.554, *d* = 1.06) or between Hard and Ramp (Ψ = −0.68, 95% CI: [−1.97, 0.60], *t* = −1.358 < *t*-critical = 2.554, *d* = 0.67).

#### 3.5.3 Level of stress

**Figure 8:**
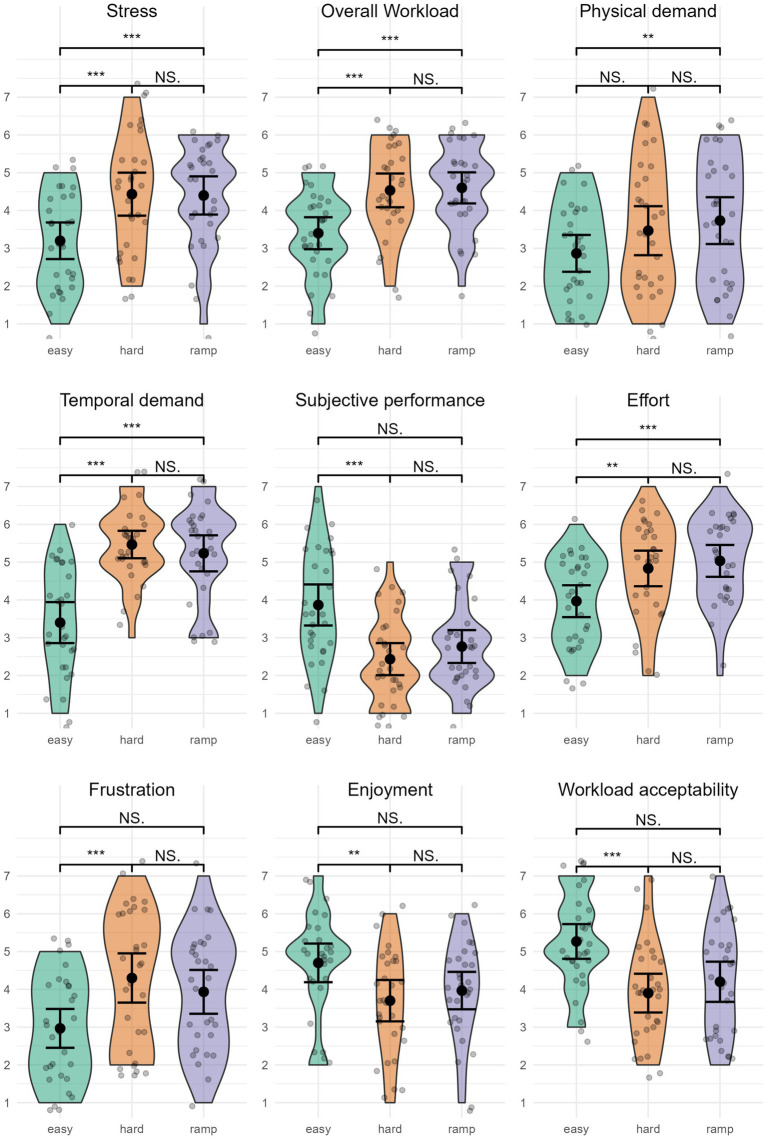
Subjective variables. ^**^p < 0.05; ^***^p < 0.01; NS., not significant. Error bars represent 95% CI assuming normality.

Level of stress (Figure 8) was highest in Hard, *M* = 4.43 (1.52), closely followed by Ramp, *M =* 4.40 (1.35). Easy had the lowest level of stress, *M =* 3.20 (1.30). The level of stress was significantly different between conditions, *χ*^2^(2) = 15.92, *p <* 0.001. Pairwise comparisons indicated a significantly higher level of stress in Ramp (*p =* 0.009, *r* = 0.54) and Hard (*p =* 0.004, *r* = 0.59) compared to Easy. There was no significant difference between Hard and Ramp (*p =* 1, *r* = 0.05).

#### 3.5.4 Overall workload

Overall workload was highest in Ramp, *M =* 4.60 (1.10), followed by Hard, *M =* 4.53 (1.20). Easy had the lowest overall workload, *M =* 3.40 (1.13). Overall workload significantly differed between conditions, *χ*^2^(2) = 19.05, *p <* 0.001. Pairwise comparisons between conditions indicated that Easy had a significantly lower overall workload than Ramp (*p =* 0.006, *r* = −0.57), and Hard (*p =* 0.003, *r* = −0.60). Overall workload was not significantly different in Ramp compared to Hard (*p =* 1, *r* = 0.04).

#### 3.5.5 TLX: physical demand

Physical demand was highest in Ramp, *M =* 3.73 (1.66), followed by Hard, *M =* 3.47 (1.74). Physical demand was lowest in Easy, *M =* 2.87 (1.31). Physical demand was significantly different between conditions, *χ*^2^(2) = 10.04, *p =* 0.007. Pairwise comparisons yielded a significant difference between Easy and Ramp (*p =* 0.035, *r* = −0.46). There was no significant difference between Easy and Hard (*p =* 0.158, r = −0.35), or Hard and Ramp (*p =* 1, *r* = −0.11).

#### 3.5.6 TLX: temporal

Temporal demand was highest in Hard, *M =* 5.47 (0.97), closely followed by Ramp, *M =* 5.23 (1.28). Easy had the lowest temporal demand, *M =* 3.40 (1.45). Temporal demand was significantly different between conditions, χ^2^(2) = 30.45, *p <* 0.001. Pairwise comparisons showed a significantly lower temporal demand in Easy compared to Ramp (*p <* 0.001, *r* = −0.72) and compared to Hard (*p <* 0.001, *r* = −0.84). There was no significant difference between Ramp and Hard (*p =* 1, *r* = 0.12).

#### 3.5.7 TLX: performance

Subjective performance was rated highest in Easy, *M =* 3.87 (1.46), followed by Ramp, *M =* 2.77 (1.17), and Hard, *M =* 2.43 (1.14). Subjective performance was significantly different between conditions, *χ*^2^(2) = 17.18, *p <* 0.001. Pairwise comparisons indicated significantly higher subjective performance in Easy than Hard (*p =* 0.003, *r* = 0.60). The comparison between Easy and Ramp achieved *p =* 0.060, *r* = 0.42. There was no significant difference between Ramp and Hard (*p =* 0.999, *r* = −0.18).

#### 3.5.8 TLX: effort

Effort was rated highest in Ramp, *M =* 5.03 (1.13), followed by Hard, *M =* 4.83 (1.26). Effort was lowest in Easy, *M =* 3.97 (1.13). Effort was significantly different between conditions, *χ*^2^(2) = 15.74, *p <* 0.001. Pairwise comparisons indicated a significantly higher effort in Ramp compared to Easy (*p =* 0.002, *r* = 0.61) and a significantly higher effort in Hard compared to Easy (*p =* 0.043, *r* = 0.45). There was no significant difference between Ramp and Hard (*p =* 1, *r* = 0.16).

#### 3.5.9 TLX: frustration

Frustration was highest in Hard, *M =* 4.30 (1.75), followed by Ramp, *M =* 3.93 (1.55). Easy had the lowest frustration, *M =* 2.97 (1.38). Frustration was significantly different between conditions, *χ*^2^(2) = 16.78, *p <* 0.001. Pairwise comparisons indicate a significantly higher frustration in Hard compared to Easy (*p =* 0.001, r = 0.65). There was no significant difference between Hard and Ramp (*p =* 0.413, *r* = −0.27), nor between Ramp and Easy (*p =* 0.117, *r* = 0.38).

#### 3.5.10 Enjoyment

Enjoyment was rated highest in Easy, *M =* 4.70 (SD = 1.37), followed by Ramp, *M* = 3.97 (1.33). Hard was rated as the least enjoyable condition, *M =* 3.70 (1.45). Enjoyment was significantly different between conditions, *χ*^2^(2) = 10.04, *p =* 0.007. Pairwise comparisons indicated that Easy was significantly more enjoyable than Hard (*p =* 0.024, *r* = 0.48), but there was no significant difference between Easy and Ramp (*p =* 0.14, *r* = 0.37) nor between Ramp and Hard (*p =* 1, *r* = −0.12).

#### 3.5.11 Workload acceptability

Workload acceptability was highest in Easy, *M =* 5.27 (1.23), followed by Ramp, *M =* 4.20 (1.42). Workload was least acceptable in Hard, *M =* 3.90 (1.37). Workload acceptability was significantly different between conditions, *χ*^2^(2) = 12.66, *p =* 0.002. Workload acceptability was significantly lower in Hard compared to Easy (*p =* 0.004, *r* = 0.60). There was no significant difference between Hard and Ramp (*p =* 0.905, *r* = −0.19). Although the comparison between Ramp and Easy achieved *p =* 0.085, *r* = 0.40, it is worth noting that their 95% Mean CI do not overlap, which is an indication of significance in the case of parametric tests.

### 3.6 EDA

There were no significant differences between conditions for nSCR (F_t_(1.84, 47.71) = 0.33, *p =* 0.70), Tonic (*t =* 0.097 *< t*-critical = 2.799), AmpSum (*t =* 2.023 *< t*-critical = 3.231), and ISCR (*t =* 2.386 *< t*-critical = 2.954). For Latency, SCR, PhasicMax, Mean, MaxDeflection, there were no significant differences between conditions (no test statistic was output).

### 3.7 Reaction time

The factorial model yielded non-significant main effects of game difficulty (*ε* = 0.875, F_t_(1.750, 45.505) = 0.207, *p =* 0.785), alarm (ε = 0.733, F_t_(2.931, 76.199) = 2.374, *p =* 0.078), and non-significant difficulty–alarm interaction effect (*ε* = 0.644, F_t_(5.148, 133.852) = 0.221, *p =* 0.956). The mixed-effects linear model corroborated these non-significant results. In other words, reaction time was not significantly different between game difficulties and alarm numbers, nor was there a significant interaction effect between difficulty and alarm number.

## 4 Discussion

All performance variables yielded significant differences between conditions (apart from Total score for the Hard-Ramp contrast), with large effect sizes. Participants performed best in Easy, followed by Ramp and Hard. This suggests our experimental manipulation was successful in creating different workloads for participants, with Easy being the least difficult, followed by Ramp and Hard.

The subjective variables further support this, providing additional details on participants’ experience of the different workloads. Hard and Ramp received similar ratings for overall workload, levels of stress, physical demand, temporal demand, effort, and frustration, which were generally significantly higher than Easy. Easy received higher ratings of valence, subjective performance, enjoyment, and workload acceptability than Hard and Ramp, but there were only significant differences between Easy and Hard. Participants reported not only higher effort and workload but also valence, enjoyment, and workload acceptability in Ramp compared to Hard. Considering that Ramp ended on a higher difficulty level than Hard, this suggests that incremental adaptation to high workload levels affects participants’ perceptions of how enjoyable and acceptable it is to be subjected to said workload, and their effort. When subjected to a high workload incrementally, participants perceive they are able to exert higher effort. Ramp is associated with more enjoyment, workload acceptability, and pleasant emotions despite higher workload. Arousal received similar ratings overall, suggesting participants were highly alert for all conditions. This could indicate that the opportunity to win a gift certificate impacted their motivation and effort in the gameplay. The enjoyment and valence ratings might indicate eustress vs. distress, suggesting more a state of eustress than distress in Ramp compared to Hard, and vice versa.

The manipulation check yielded mixed fNIRS results, HbR suggesting higher cognitive load in Hard1, and HbO suggesting higher cognitive load in Easy1. This is surprising as we expected increased cognitive load in Hard1 compared to Easy1 (H1). This could indicate that Easy1 was more cognitively demanding than Hard1, that Hard1 was so difficult that participants were unable to recruit as many neuronal resources as in Easy1, or that mental fatigue or cognitive disengagement occurred in Hard1 (compared to Easy1). The competitive element of possibly attaining a high-value gift certificate could also have influenced participants. For EEG, the occipital Theta power increase indicates that Hard1 was more cognitively demanding than Easy1, supporting H1 and corroborating existing literature (Antonenko et al., 2010; Borghini et al., 2014; Chikhi et al., 2022; Hamann and Carstengerdes, 2022). The occipital Alpha power increase further suggests mental fatigue occurred in Hard1 compared to Easy1. It is reasonable to assume mental fatigue would be greater in Hard1 compared to Easy1, and this could explain the fNIRS results, should mental fatigue have led to cognitive disengagement.

The fNIRS contrasts comparing the first and last minute of constant load conditions evidenced higher cognitive load at the end (for both Easy and Hard), supporting the hypothesis of a temporal effect of workload (H2). Despite equal workload, participants’ cognitive load increases, perhaps due to the need for sustained attention. For EEG, the right parietal Delta power increase indicates the presence of mental fatigue in Easy4 compared to Easy1, corroborating the fNIRS results and existing literature (Borghini et al., 2014). The non-significant EEG variables for the Hard4-Hard1 contrast could indicate that the workload was too high to be able to distill different levels of mental fatigue.

For Ramp contrasts, those contrasts comparing two adjacent minutes exhibit minor differences, as expected with the incremental difficulty level increases. Adjacent contrasts at the end (Ramp3/Ramp4) exhibit greater differences than adjacent contrasts at the start (Ramp1/Ramp2), as expected given the lower difficulty level at the start and the expectation of mental fatigue occurring sometime in the end. Contrasts comparing non-adjacent minutes exhibit greater differences as expected. The results suggest higher cognitive activation at the start of gameplay, and as difficulty level increases, cognitive activation decreases, perhaps because it becomes more difficult to recruit sufficient neuronal resources. Based on these contrasts alone, it is difficult to ascertain whether participants cognitively disengaged or mentally fatigued at the gameplay’s end (i.e., in Ramp4 or Ramp3), but that is a possibility. Participant observations made by the experimenter would support that. For EEG, none of the electrodes were sensitive enough to discriminate between contrasts within the Ramp.

In this context, EEG appears to be less sensitive to small changes in cognitive workload than fNIRS. Changing discrimination ability with difficulty level was evidenced by Hamann and Carstengerdes (2022), EEG could not discriminate between lower workload levels, while fNIRS could not discriminate between higher workload levels. The authors proposed an alternative explanation, that participants reached their cognitive resource limit, making it difficult to distinguish between higher workload levels. Similarly, in this study, there were minor differences between the high difficulty level contrasts of Ramp. This could be interpreted as participants reaching or getting closer to a cognitive threshold. This notion of such a cognitive threshold is supported by others, that is, when task difficulty level exceeds a certain threshold, activation decreases compared to lower loads (Baker et al., 2018; Meidenbauer et al., 2021; Parent et al., 2019). An explanation could be that task demands exceed participants’ cognitive capacity, they mentally disengage from the task, potentially because of failing to recruit sufficient neuronal resources (Meidenbauer et al., 2021), or the induced stress and influencing selective attention (Baker et al., 2018). Mental disengagement could certainly explain our results for the Hard1-Easy1 contrast. Identification of individual upper cognitive thresholds ought to be focused on in further research.

ECG variables HFpeak [Hz] and HFpow [log] were significantly lower in Ramp2 compared to Ramp1, indicating that Ramp2 elicited a higher stress response than Ramp, partially supporting H1: there is an effect of difficulty level on stress response. However, this effect emerged only when comparing incremental difficulty level increases over time, i.e., in Ramp, and not for Hard-Easy comparisons, suggesting that for constant difficulty, high and low difficulties cannot be significantly discriminated with ECG, at least within this experimental paradigm where participants had financial incentive. Furthermore, apart from those variables (HFpeak and HFpow), no ECG variables yielded significant differences between conditions, indicating no difference in participants’ physiological stress response between conditions, supporting the rejection of H2. There were no significant differences between any EDA variables.

The non-significant differences in stress could be explained by several factors. Subjectively evaluated arousal received similar ratings overall, and was not significantly different between Easy and Hard, which supports undistinguishable physiologically measured arousal (ECG). Participants were financially incentivized to perform well, which could be achieved through maintaining high arousal and exposure to alarm sounds. The alarm sound could have acted as a noise stressor, potentially contributing to high arousal, in turn contributing to the lack of differences in ECG and EDA variables. It could have been interesting to see whether results were similar had we not included the AUT and/or participants not been incentivized in the same way. Our non-significant ECG variables partially corroborate other research. Parent et al. (2019) were not able to classify stress with ECG variables alone significantly, but needed additional fNIRS variables to classify stress. However, ECG variables alone were able to classify mental workload (Parent et al., 2019). The authors suggest that fNIRS and ECG in combination best disentangle the concepts of mental workload and stress (Parent et al., 2019). Others researchers found that only some ECG variables were sensitive enough to significantly discriminate between workload levels (Cinaz et al., 2013; Wulvik et al., 2019). Although the physiological stress response was not different between Easy and Hard, the subjective ratings indicated participants experienced a more unpleasant or negative affective state in Hard (valence, enjoyment) while Easy was more enjoyable and associated with more pleasant or positive affective state, and the valence difference had a large effect size. Taken together, these results further support the notion of eustress and destress, i.e., although the physiological stress response is not different between Easy and Hard, they are perceived differently by participants.

### 4.1 Limitations and implications for future research

Mental demand (TLX dimension) was not collected due to an error. There were limitations related to the use of Affect Grid. Several participants appeared to struggle when attempting to understand the affect grid’s interface and how to fill it out, filling the form out incorrectly, yielding missing data. It might be better to split the Affect Grid into two separate questions in the future experiments. The experiment was conducted during COVID-19 (May and June 2021), necessitating infection control measures, resulting in approximately 50% dropout from participants reporting initial interest via email. We expect a greater sample size had the experiment not been conducted during COVID-19. A larger sample size would have increased the results’ generalizability. Due to the nature of the different neuroimaging and physiology sensors, it was not possible to analyze all neurophysiological variables in precisely the same manner within this experimental procedure. This, together with the experimental design, could have impacted our results. These limitations could have influenced the non-significant ECG and EDA variables. Our fNIRS optodes and EEG electrodes could not be placed in the same location. Our multimodal data collection likely increased the data loss magnitude compared to if we collected only one modality. In addition to Tetris’s perhaps inherent competitive aspects, participants were additionally incentivized to perform better than other participants to win a gift certificate. This could have contributed to the high physiological and subjective arousal in all conditions. Therefore, it could have been valuable to estimate participants’ competitiveness as a control variable, e.g., self-reported competitiveness. Investigating the temporal effect of workload with fNIRS is difficult due to the ceiling effects of the hemodynamic response in blocks over 60 s. Future research could aim to discriminate between the temporal effect of workload and the ceiling effects of the hemodynamic response. This experiment did not separate workload and stress properly, which future experiments ought to. While workload varied as intended, we likely had high physiological stress for all conditions, possibly due to the ART and financial incentive. The ART did not yield any differences, likely due to its implementation or perhaps an incorrect number of alarms, and consequently, it acted as an additional stressor. We would not have included the ART or financial incentive were we to set up a similar experiment again. However, we believe an ART could be suitable for objectively measuring workload in experiments with a less intense primary task. The different sensor data cannot be analyzed on the same timescale, which is an inherent sensor problem. The study was therefore differently powered for different variables. Nevertheless, in future experiments we recommend setting conditions to better facilitate a similar analysis process across sensors (provided that is an aim in itself, of course). Although EEG provided complementary data to fNIRS, we are not as confident of EEG’s data quality as that of fNIRS’. We expect a different EEG sensor, perhaps high-density, with a different application procedure or electrode tips, would provide higher quality data. Finally, it is essential to be mindful about sensor selection, consider the sensor’s validity for the construct sought to measure, and which sensor provides more/less data.

While Tetris gameplays and the complex, dynamically changing environments described initially have similar features, they are not the same. It could be expensive to run multimodal studies in highly ecologically valid scenarios. When investigating if and when neurophysiological tools can discern workloads/stress, Tetris provides a practical, scalable, and less costly method to measure cognitive load in controlled environments. After suitable sensors have been identified, experiments with high ecological validity can be undertaken, reducing the overall research cost.

## 5 Conclusion

This study investigated the relationship between workload, performance, and the human mental and physical state by measuring cognitive load and stress in a complex, dynamically changing environment (Tetris gameplay) through fNIRS, EEG, ECG, EDA, performance, and subjective self-reports. Performance variables evidenced a successful experimental manipulation: Hard was most difficult, followed by Ramp and Easy. As a result, fNIRS, EEG, and ECG data partially supported increased cognitive load and stress with increasing workload. We also found evidence of reduced fNIRS activation for higher workloads, possibly due to mental fatigue or disengagement, corroborating literature. We furthermore found evidence for a temporal effect of workload on cognitive load (i.e., irrespective of difficulty), fNIRS yielding higher activation and EEG yielding mental fatigue with increasing time while difficulty was held constant. Despite large effects on cognitive load, the present experimental paradigm yielded little difference in physiological stress response between conditions. Simultaneously, subjective data indicated that participants experienced Hard as more unpleasant, and Easy as more pleasant and enjoyable, with a large effect size. Participants’ different perceptions of conditions (i.e., differing affective state), while experiencing undistinguishable physiological stress, could serve as evidence for a state of eustress and destress. Finally, not all neurophysiological variables were able to discern different workloads. As such, our multimodal data collection provided complementary data, providing a more complete picture, which aided in the interpretation of results.

## Data Availability

The datasets presented in this study can be found in online repositories. The names of the repository/repositories and accession number(s) can be found at: OSF https://doi.org/10.17605/OSF.IO/M47WH.

## Footnotes

^1^The dimension Mental Demand was not collected due to an error.

^2^In a few cases, the total recording length was shorter than 4 min (e.g., 3:53). In this case, the two slices overlapped slightly to attain the 2-min duration minimum.
