# Supplementary material for: fNIRS, EEG, ECG, and GSR reveal an effect of complex, dynamically changing environments on cognitive load, affective state, and performance, but not physiological stress
Source: Front Hum Neurosci. 2025 Jun 2;19:1459653. doi: 10.3389/fnhum.2025.1459653 (PMC12171126; doi:10.3389/fnhum.2025.1459653)
Supplement: Supplementary file 2 [file Data_Sheet_1.docx]

Supplementary Material

# Supplementary fNIRS results

## Manipulation check – Supplementary statistics

Table 1 Contrast statistics for Hard1-Easy1.

| source | detector | type | beta | se | tstat | dfe | p | q | min Discoverable Change | Relative Power |
| --- | --- | --- | --- | --- | --- | --- | --- | --- | --- | --- |
| 1 | 1 | hbo | -41.996 | 5.434 | -7.728 | 348 | 0.0000 | 0.0000 | 13.542 | 0.310 |
| 1 | 1 | hbr | 5.720 | 3.327 | 1.719 | 348 | 0.0864 | 0.1729 | 8.290 | 0.506 |
| 1 | 2 | hbo | -49.532 | 7.539 | -6.571 | 348 | 0.0000 | 0.0000 | 18.785 | 0.223 |
| 1 | 2 | hbr | 4.646 | 4.169 | 1.115 | 348 | 0.2658 | 0.4430 | 10.388 | 0.404 |
| 2 | 1 | hbo | -10.115 | 3.665 | -2.760 | 348 | 0.0061 | 0.0305 | 9.133 | 0.459 |
| 2 | 1 | hbr | -5.773 | 2.279 | -2.533 | 348 | 0.0118 | 0.0470 | 5.680 | 0.738 |
| 2 | 3 | hbo | -7.159 | 5.495 | -1.303 | 348 | 0.1935 | 0.3518 | 13.693 | 0.306 |
| 2 | 3 | hbr | -6.724 | 3.042 | -2.210 | 348 | 0.0278 | 0.0740 | 7.581 | 0.553 |
| 3 | 2 | hbo | -2.336 | 2.904 | -0.805 | 348 | 0.4217 | 0.5622 | 7.235 | 0.579 |
| 3 | 2 | hbr | -2.829 | 1.682 | -1.682 | 348 | 0.0935 | 0.1781 | 4.192 | 1.000 |
| 3 | 3 | hbo | -5.295 | 5.917 | -0.895 | 348 | 0.3715 | 0.5307 | 14.745 | 0.284 |
| 3 | 3 | hbr | -1.491 | 3.528 | -0.423 | 348 | 0.6729 | 0.8411 | 8.791 | 0.477 |
| 3 | 4 | hbo | -3.774 | 3.226 | -1.170 | 348 | 0.2429 | 0.4225 | 8.040 | 0.521 |
| 3 | 4 | hbr | -6.666 | 2.231 | -2.988 | 348 | 0.0030 | 0.0200 | 5.559 | 0.754 |
| 4 | 2 | hbo | -20.263 | 4.711 | -4.301 | 348 | 0.0000 | 0.0002 | 11.739 | 0.357 |
| 4 | 2 | hbr | -1.133 | 3.515 | -0.322 | 348 | 0.7473 | 0.8826 | 8.760 | 0.479 |
| 4 | 4 | hbo | 0.318 | 3.859 | 0.082 | 348 | 0.9344 | 0.9583 | 9.617 | 0.436 |
| 4 | 4 | hbr | -5.295 | 2.373 | -2.231 | 348 | 0.0263 | 0.0740 | 5.914 | 0.709 |
| 4 | 5 | hbo | 0.034 | 6.906 | 0.005 | 348 | 0.9961 | 0.9961 | 17.209 | 0.244 |
| 4 | 5 | hbr | -3.244 | 4.272 | -0.759 | 348 | 0.4481 | 0.5782 | 10.646 | 0.394 |
| 5 | 3 | hbo | -13.872 | 3.213 | -4.318 | 348 | 0.0000 | 0.0002 | 8.006 | 0.524 |
| 5 | 3 | hbr | -0.275 | 2.103 | -0.131 | 348 | 0.8961 | 0.9433 | 5.240 | 0.800 |
| 5 | 4 | hbo | -8.962 | 3.180 | -2.818 | 348 | 0.0051 | 0.0292 | 7.925 | 0.529 |
| 5 | 4 | hbr | 3.883 | 2.108 | 1.842 | 348 | 0.0663 | 0.1434 | 5.252 | 0.798 |
| 5 | 6 | hbo | -0.563 | 3.441 | -0.164 | 348 | 0.8702 | 0.9433 | 8.574 | 0.489 |
| 5 | 6 | hbr | -6.750 | 2.710 | -2.491 | 348 | 0.0132 | 0.0481 | 6.754 | 0.621 |
| 6 | 4 | hbo | -7.809 | 3.245 | -2.406 | 348 | 0.0166 | 0.0512 | 8.086 | 0.518 |
| 6 | 4 | hbr | -2.268 | 2.519 | -0.900 | 348 | 0.3687 | 0.5307 | 6.278 | 0.668 |
| 6 | 5 | hbo | -2.776 | 2.588 | -1.073 | 348 | 0.2842 | 0.4547 | 6.450 | 0.650 |
| 6 | 5 | hbr | 0.503 | 1.736 | 0.290 | 348 | 0.7722 | 0.8826 | 4.327 | 0.969 |
| 6 | 6 | hbo | -3.503 | 4.172 | -0.840 | 348 | 0.4017 | 0.5540 | 10.395 | 0.403 |
| 6 | 6 | hbr | 0.867 | 2.850 | 0.304 | 348 | 0.7612 | 0.8826 | 7.102 | 0.590 |
| 7 | 5 | hbo | 0.561 | 4.131 | 0.136 | 348 | 0.8921 | 0.9433 | 10.295 | 0.407 |
| 7 | 5 | hbr | -6.286 | 3.125 | -2.011 | 348 | 0.0451 | 0.1126 | 7.787 | 0.538 |
| 7 | 7 | hbo | 13.939 | 5.766 | 2.418 | 348 | 0.0161 | 0.0512 | 14.368 | 0.292 |
| 7 | 7 | hbr | -8.661 | 4.531 | -1.911 | 348 | 0.0568 | 0.1336 | 11.292 | 0.371 |
| 8 | 6 | hbo | -20.883 | 3.717 | -5.618 | 348 | 0.0000 | 0.0000 | 9.264 | 0.453 |
| 8 | 6 | hbr | -6.667 | 2.534 | -2.631 | 348 | 0.0089 | 0.0395 | 6.314 | 0.664 |
| 8 | 7 | hbo | -4.470 | 4.268 | -1.047 | 348 | 0.2956 | 0.4548 | 10.635 | 0.394 |
| 8 | 7 | hbr | -4.522 | 2.471 | -1.830 | 348 | 0.0681 | 0.1434 | 6.158 | 0.681 |

## Contrasting 1st and 4^th^ minute in constant load conditions – Supplementary statistics

Table 2 Contrast statistics for Easy4-Easy1

| source | detector | type | beta | se | tstat | dfe | p | q | min Discoverable Change | Relative Power |
| --- | --- | --- | --- | --- | --- | --- | --- | --- | --- | --- |
| 1 | 1 | hbo | 11.267 | 5.375 | 2.096 | 348 | 0.0368 | 0.1161 | 13.394 | 0.294 |
| 1 | 1 | hbr | 7.475 | 3.198 | 2.338 | 348 | 0.0200 | 0.0726 | 7.969 | 0.493 |
| 1 | 2 | hbo | 14.621 | 7.418 | 1.971 | 348 | 0.0495 | 0.1283 | 18.485 | 0.213 |
| 1 | 2 | hbr | 18.341 | 4.017 | 4.566 | 348 | 0.0000 | 0.0001 | 10.010 | 0.393 |
| 2 | 1 | hbo | -5.697 | 3.605 | -1.580 | 348 | 0.1149 | 0.2362 | 8.984 | 0.438 |
| 2 | 1 | hbr | 2.677 | 2.221 | 1.205 | 348 | 0.2289 | 0.4161 | 5.534 | 0.711 |
| 2 | 3 | hbo | -2.168 | 5.471 | -0.396 | 348 | 0.6921 | 0.8389 | 13.633 | 0.288 |
| 2 | 3 | hbr | 7.083 | 2.971 | 2.384 | 348 | 0.0176 | 0.0706 | 7.402 | 0.531 |
| 3 | 2 | hbo | 15.191 | 2.844 | 5.341 | 348 | 0.0000 | 0.0000 | 7.088 | 0.555 |
| 3 | 2 | hbr | 0.321 | 1.578 | 0.203 | 348 | 0.8389 | 0.9041 | 3.932 | 1.000 |
| 3 | 3 | hbo | 19.077 | 5.779 | 3.301 | 348 | 0.0011 | 0.0077 | 14.400 | 0.273 |
| 3 | 3 | hbr | 9.337 | 3.319 | 2.813 | 348 | 0.0052 | 0.0317 | 8.272 | 0.475 |
| 3 | 4 | hbo | 2.586 | 3.368 | 0.768 | 348 | 0.4432 | 0.6220 | 8.393 | 0.468 |
| 3 | 4 | hbr | 0.022 | 1.910 | 0.011 | 348 | 0.9910 | 0.9910 | 4.760 | 0.826 |
| 4 | 2 | hbo | -0.818 | 4.807 | -0.170 | 348 | 0.8650 | 0.9041 | 11.978 | 0.328 |
| 4 | 2 | hbr | 5.080 | 2.898 | 1.753 | 348 | 0.0805 | 0.1841 | 7.222 | 0.544 |
| 4 | 4 | hbo | 1.947 | 3.876 | 0.502 | 348 | 0.6158 | 0.7946 | 9.658 | 0.407 |
| 4 | 4 | hbr | -8.349 | 2.441 | -3.420 | 348 | 0.0007 | 0.0056 | 6.084 | 0.646 |
| 4 | 5 | hbo | 3.664 | 6.879 | 0.533 | 348 | 0.5946 | 0.7905 | 17.143 | 0.229 |
| 4 | 5 | hbr | -2.280 | 4.232 | -0.539 | 348 | 0.5903 | 0.7905 | 10.545 | 0.373 |
| 5 | 3 | hbo | 1.742 | 3.189 | 0.546 | 348 | 0.5853 | 0.7905 | 7.946 | 0.495 |
| 5 | 3 | hbr | -2.072 | 2.023 | -1.024 | 348 | 0.3064 | 0.4806 | 5.040 | 0.780 |
| 5 | 4 | hbo | 1.645 | 3.158 | 0.521 | 348 | 0.6027 | 0.7905 | 7.870 | 0.500 |
| 5 | 4 | hbr | 2.291 | 2.002 | 1.144 | 348 | 0.2533 | 0.4296 | 4.988 | 0.788 |
| 5 | 6 | hbo | 1.039 | 3.414 | 0.304 | 348 | 0.7610 | 0.9041 | 8.509 | 0.462 |
| 5 | 6 | hbr | 0.642 | 2.668 | 0.241 | 348 | 0.8099 | 0.9041 | 6.649 | 0.591 |
| 6 | 4 | hbo | 2.652 | 3.222 | 0.823 | 348 | 0.4110 | 0.6089 | 8.030 | 0.490 |
| 6 | 4 | hbr | 5.372 | 2.490 | 2.157 | 348 | 0.0317 | 0.1077 | 6.205 | 0.634 |
| 6 | 5 | hbo | -0.629 | 2.541 | -0.248 | 348 | 0.8047 | 0.9041 | 6.331 | 0.621 |
| 6 | 5 | hbr | -0.782 | 1.694 | -0.462 | 348 | 0.6446 | 0.8057 | 4.220 | 0.932 |
| 6 | 6 | hbo | -3.372 | 4.240 | -0.795 | 348 | 0.4270 | 0.6182 | 10.566 | 0.372 |
| 6 | 6 | hbr | 3.854 | 2.873 | 1.341 | 348 | 0.1807 | 0.3442 | 7.160 | 0.549 |
| 7 | 5 | hbo | 8.536 | 4.156 | 2.054 | 348 | 0.0407 | 0.1175 | 10.356 | 0.380 |
| 7 | 5 | hbr | -4.957 | 3.261 | -1.520 | 348 | 0.1294 | 0.2589 | 8.126 | 0.484 |
| 7 | 7 | hbo | 16.015 | 5.786 | 2.768 | 348 | 0.0059 | 0.0317 | 14.417 | 0.273 |
| 7 | 7 | hbr | -8.540 | 4.589 | -1.861 | 348 | 0.0636 | 0.1590 | 11.437 | 0.344 |
| 8 | 6 | hbo | -4.093 | 3.693 | -1.108 | 348 | 0.2685 | 0.4383 | 9.203 | 0.427 |
| 8 | 6 | hbr | 1.006 | 2.488 | 0.404 | 348 | 0.6862 | 0.8389 | 6.199 | 0.634 |
| 8 | 7 | hbo | 0.716 | 4.109 | 0.174 | 348 | 0.8618 | 0.9041 | 10.239 | 0.384 |
| 8 | 7 | hbr | 0.488 | 2.488 | 0.196 | 348 | 0.8445 | 0.9041 | 6.200 | 0.634 |

Table 3 Contrast statistics for Hard4-Hard1

| source | detector | type | beta | se | tstat | dfe | p | q | min Discoverable Change | Relative Power |
| --- | --- | --- | --- | --- | --- | --- | --- | --- | --- | --- |
| 1 | 1 | hbo | 11.267 | 5.375 | 2.096 | 348 | 0.0368 | 0.1161 | 13.394 | 0.294 |
| 1 | 1 | hbr | 7.475 | 3.198 | 2.338 | 348 | 0.0200 | 0.0726 | 7.969 | 0.493 |
| 1 | 2 | hbo | 14.621 | 7.418 | 1.971 | 348 | 0.0495 | 0.1283 | 18.485 | 0.213 |
| 1 | 2 | hbr | 18.341 | 4.017 | 4.566 | 348 | 0.0000 | 0.0001 | 10.010 | 0.393 |
| 2 | 1 | hbo | -5.697 | 3.605 | -1.580 | 348 | 0.1149 | 0.2362 | 8.984 | 0.438 |
| 2 | 1 | hbr | 2.677 | 2.221 | 1.205 | 348 | 0.2289 | 0.4161 | 5.534 | 0.711 |
| 2 | 3 | hbo | -2.168 | 5.471 | -0.396 | 348 | 0.6921 | 0.8389 | 13.633 | 0.288 |
| 2 | 3 | hbr | 7.083 | 2.971 | 2.384 | 348 | 0.0176 | 0.0706 | 7.402 | 0.531 |
| 3 | 2 | hbo | 15.191 | 2.844 | 5.341 | 348 | 0.0000 | 0.0000 | 7.088 | 0.555 |
| 3 | 2 | hbr | 0.321 | 1.578 | 0.203 | 348 | 0.8389 | 0.9041 | 3.932 | 1.000 |
| 3 | 3 | hbo | 19.077 | 5.779 | 3.301 | 348 | 0.0011 | 0.0077 | 14.400 | 0.273 |
| 3 | 3 | hbr | 9.337 | 3.319 | 2.813 | 348 | 0.0052 | 0.0317 | 8.272 | 0.475 |
| 3 | 4 | hbo | 2.586 | 3.368 | 0.768 | 348 | 0.4432 | 0.6220 | 8.393 | 0.468 |
| 3 | 4 | hbr | 0.022 | 1.910 | 0.011 | 348 | 0.9910 | 0.9910 | 4.760 | 0.826 |
| 4 | 2 | hbo | -0.818 | 4.807 | -0.170 | 348 | 0.8650 | 0.9041 | 11.978 | 0.328 |
| 4 | 2 | hbr | 5.080 | 2.898 | 1.753 | 348 | 0.0805 | 0.1841 | 7.222 | 0.544 |
| 4 | 4 | hbo | 1.947 | 3.876 | 0.502 | 348 | 0.6158 | 0.7946 | 9.658 | 0.407 |
| 4 | 4 | hbr | -8.349 | 2.441 | -3.420 | 348 | 0.0007 | 0.0056 | 6.084 | 0.646 |
| 4 | 5 | hbo | 3.664 | 6.879 | 0.533 | 348 | 0.5946 | 0.7905 | 17.143 | 0.229 |
| 4 | 5 | hbr | -2.280 | 4.232 | -0.539 | 348 | 0.5903 | 0.7905 | 10.545 | 0.373 |
| 5 | 3 | hbo | 1.742 | 3.189 | 0.546 | 348 | 0.5853 | 0.7905 | 7.946 | 0.495 |
| 5 | 3 | hbr | -2.072 | 2.023 | -1.024 | 348 | 0.3064 | 0.4806 | 5.040 | 0.780 |
| 5 | 4 | hbo | 1.645 | 3.158 | 0.521 | 348 | 0.6027 | 0.7905 | 7.870 | 0.500 |
| 5 | 4 | hbr | 2.291 | 2.002 | 1.144 | 348 | 0.2533 | 0.4296 | 4.988 | 0.788 |
| 5 | 6 | hbo | 1.039 | 3.414 | 0.304 | 348 | 0.7610 | 0.9041 | 8.509 | 0.462 |
| 5 | 6 | hbr | 0.642 | 2.668 | 0.241 | 348 | 0.8099 | 0.9041 | 6.649 | 0.591 |
| 6 | 4 | hbo | 2.652 | 3.222 | 0.823 | 348 | 0.4110 | 0.6089 | 8.030 | 0.490 |
| 6 | 4 | hbr | 5.372 | 2.490 | 2.157 | 348 | 0.0317 | 0.1077 | 6.205 | 0.634 |
| 6 | 5 | hbo | -0.629 | 2.541 | -0.248 | 348 | 0.8047 | 0.9041 | 6.331 | 0.621 |
| 6 | 5 | hbr | -0.782 | 1.694 | -0.462 | 348 | 0.6446 | 0.8057 | 4.220 | 0.932 |
| 6 | 6 | hbo | -3.372 | 4.240 | -0.795 | 348 | 0.4270 | 0.6182 | 10.566 | 0.372 |
| 6 | 6 | hbr | 3.854 | 2.873 | 1.341 | 348 | 0.1807 | 0.3442 | 7.160 | 0.549 |
| 7 | 5 | hbo | 8.536 | 4.156 | 2.054 | 348 | 0.0407 | 0.1175 | 10.356 | 0.380 |
| 7 | 5 | hbr | -4.957 | 3.261 | -1.520 | 348 | 0.1294 | 0.2589 | 8.126 | 0.484 |
| 7 | 7 | hbo | 16.015 | 5.786 | 2.768 | 348 | 0.0059 | 0.0317 | 14.417 | 0.273 |
| 7 | 7 | hbr | -8.540 | 4.589 | -1.861 | 348 | 0.0636 | 0.1590 | 11.437 | 0.344 |
| 8 | 6 | hbo | -4.093 | 3.693 | -1.108 | 348 | 0.2685 | 0.4383 | 9.203 | 0.427 |
| 8 | 6 | hbr | 1.006 | 2.488 | 0.404 | 348 | 0.6862 | 0.8389 | 6.199 | 0.634 |
| 8 | 7 | hbo | 0.716 | 4.109 | 0.174 | 348 | 0.8618 | 0.9041 | 10.239 | 0.384 |
| 8 | 7 | hbr | 0.488 | 2.488 | 0.196 | 348 | 0.8445 | 0.9041 | 6.200 | 0.634 |

## Contrasts within RampGame – Supplementary figures and statistics

| 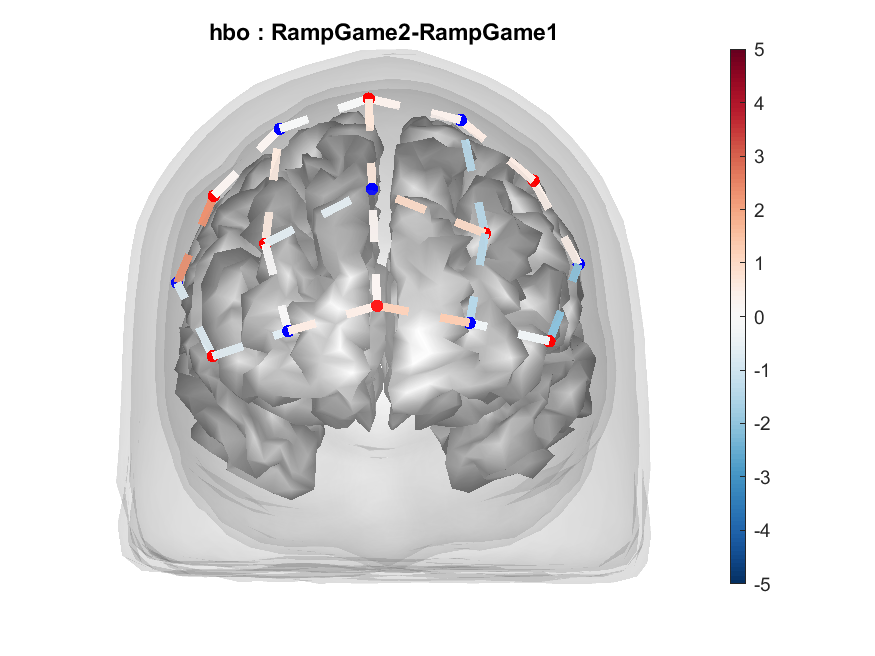 | 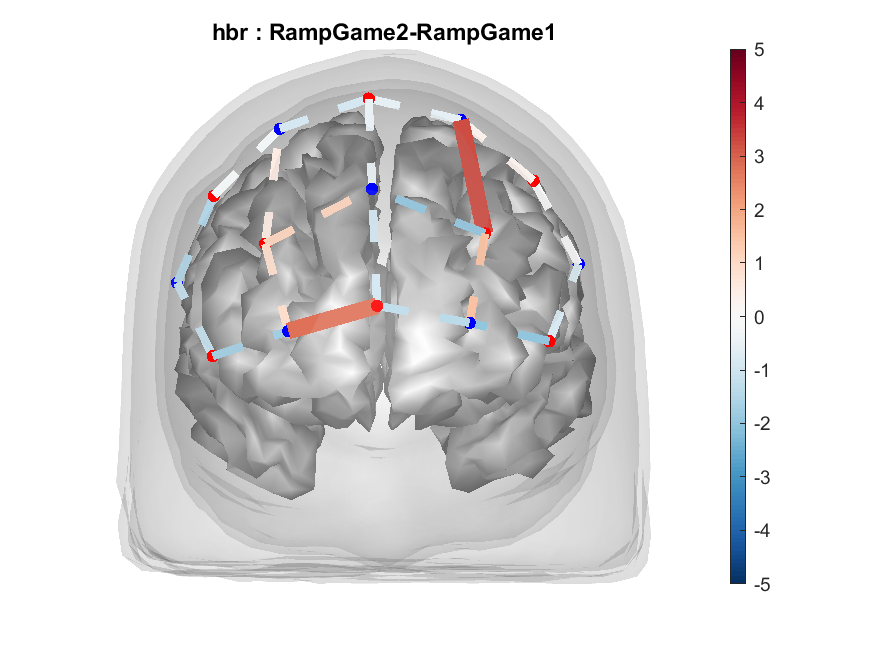 |
| --- | --- |
| 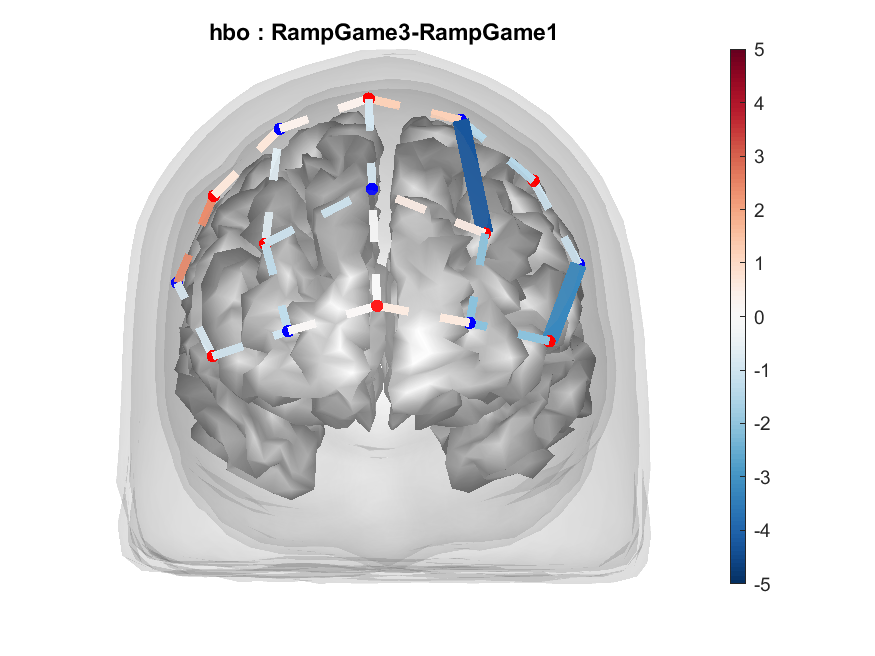 | 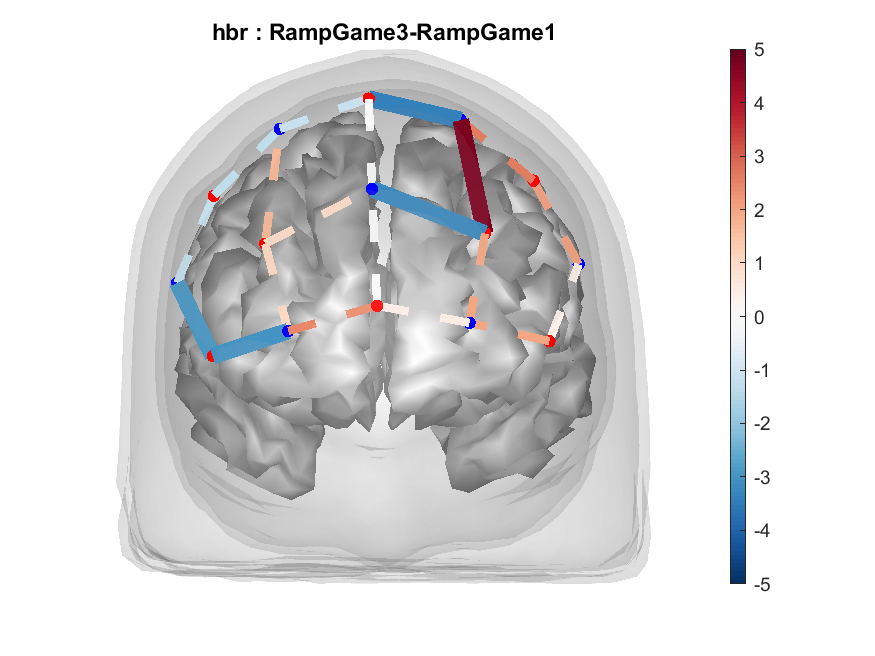 |
| 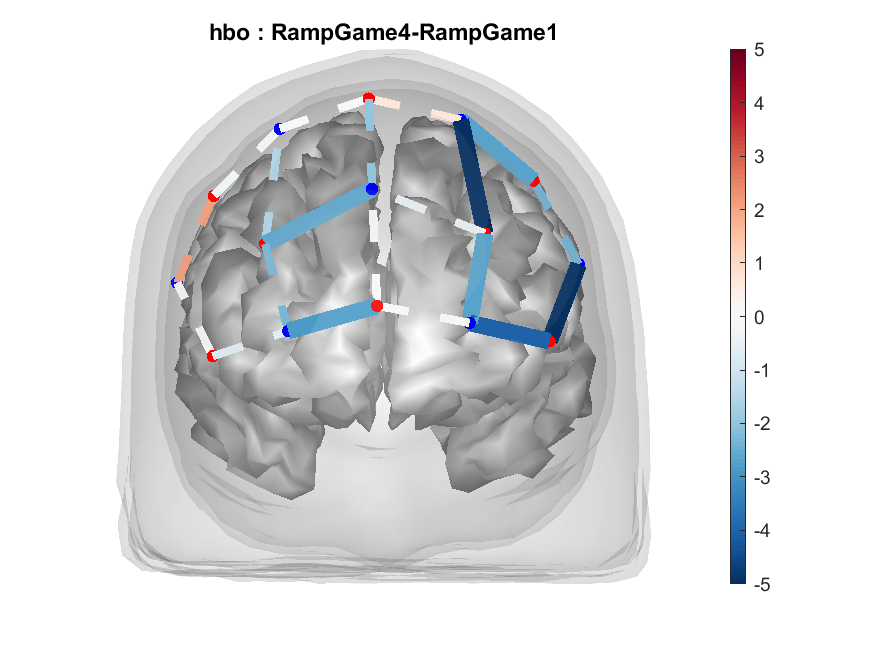 | 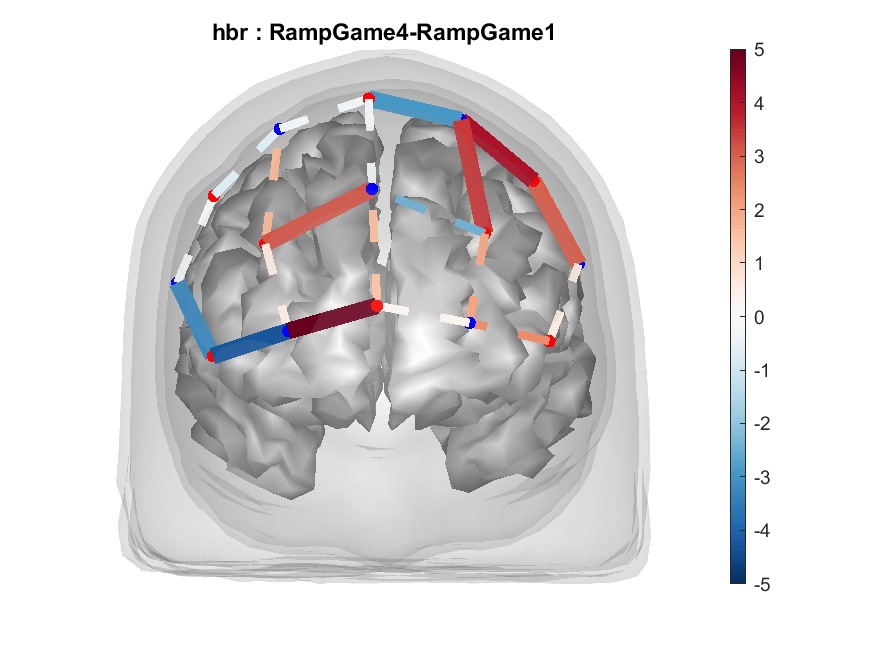 |
| 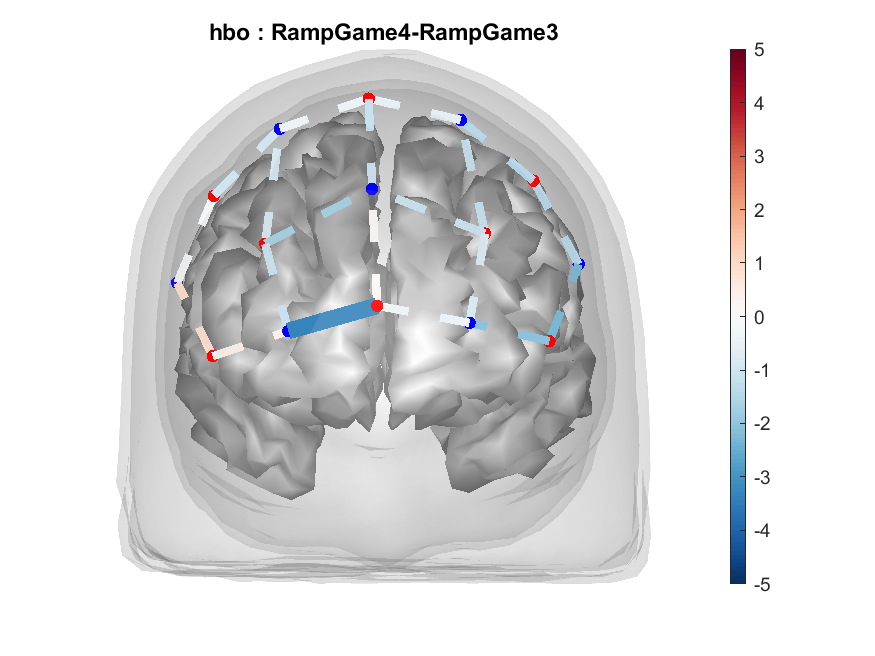 | 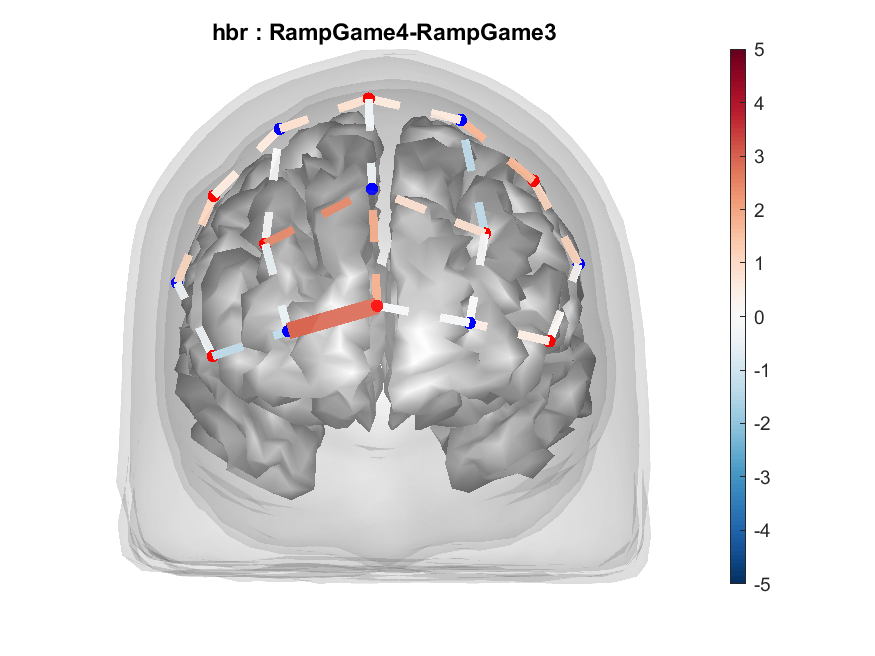 |
| 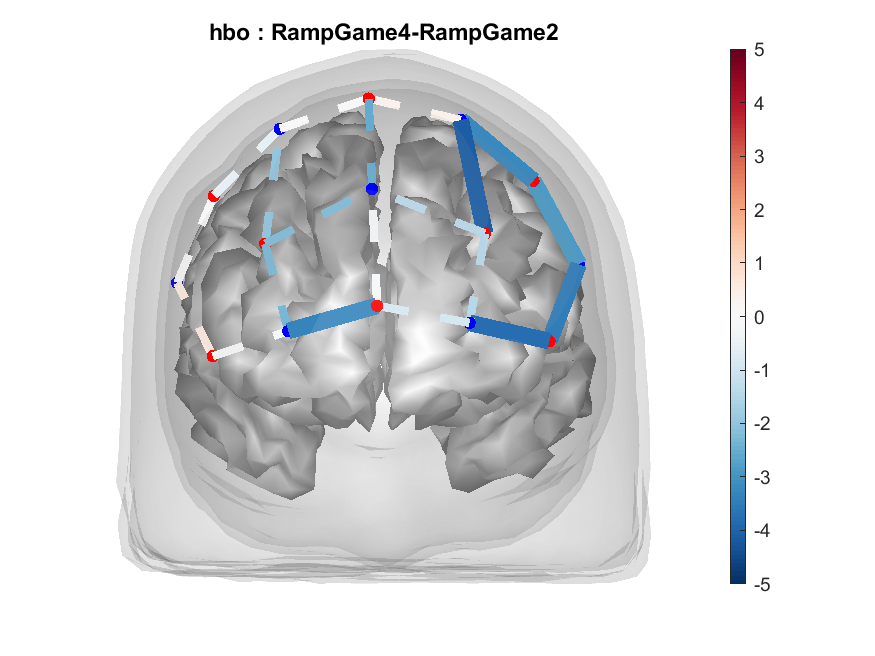 | 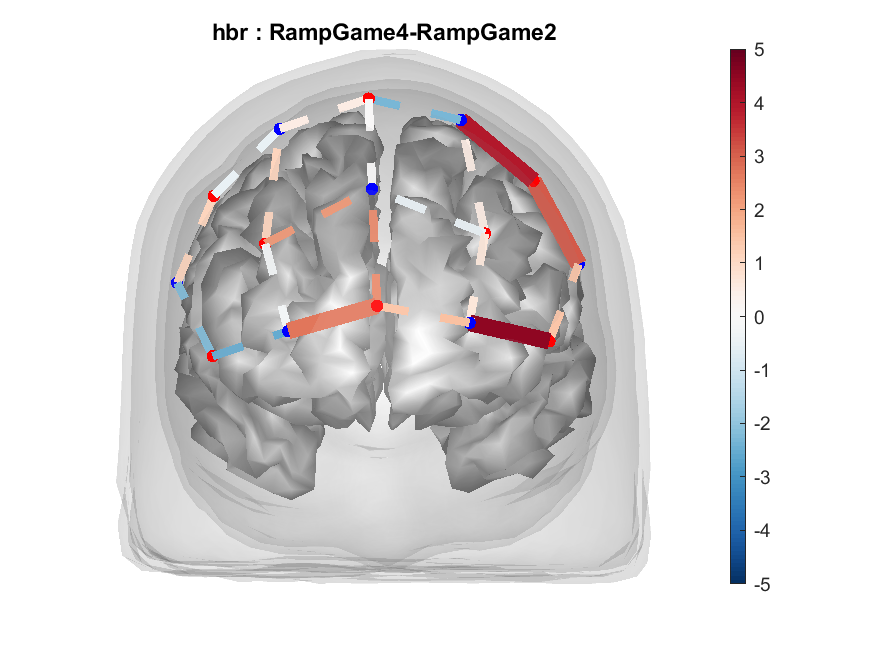 |
| 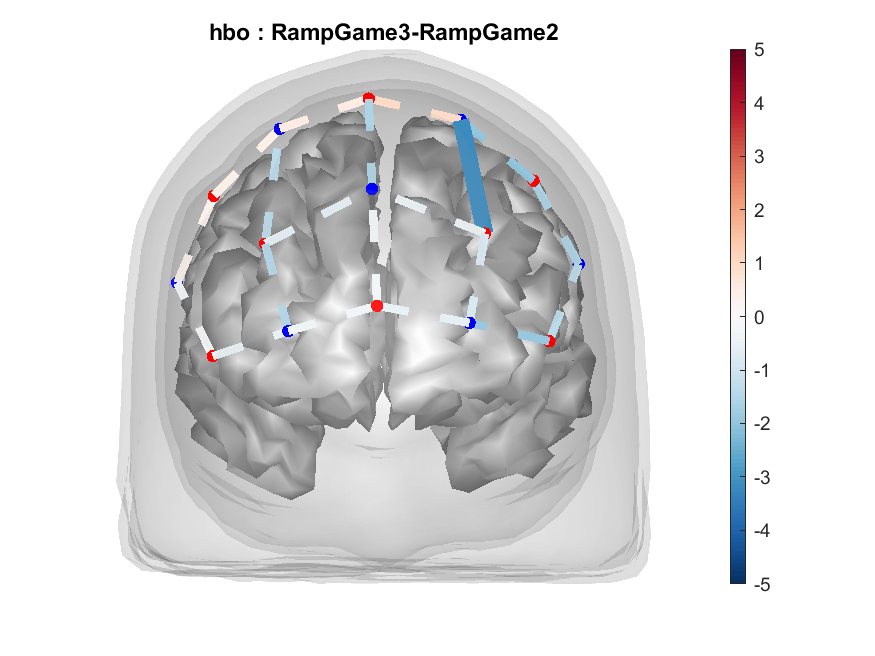 | 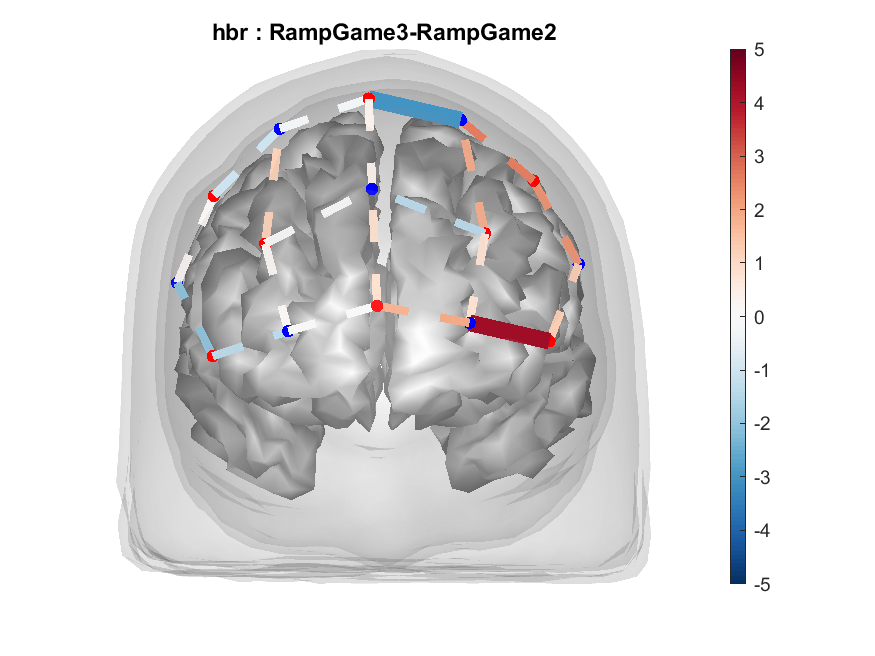 |
| Figure 1 Contrasts comparing the minutes within Ramp. The color bar represents the t-statistic scaled to [-5,5] with full red/blue lines indicating statistically significant increase/decrease at an FDR-corrected p-value q<0.05. The t-statistic is plotted onto the Colin27 atlas using NIRS Brain AnalyzIR toolbox (Santosa et al., 2018). HbO to the left, HbR to the right. | |

Table 4 Contrast statistics for Ramp contrasts

| source | detector | type | contrast | beta | se | tstat | dfe | p | q | min Discoverable Change | Relative Power |
| --- | --- | --- | --- | --- | --- | --- | --- | --- | --- | --- | --- |
| 1 | 1 | hbo | RampGame4-RampGame3 | -6.768 | 4.774 | -1.418 | 348 | 0.1572 | 0.3811 | 11.897 | 0.306 |
| 1 | 1 | hbo | RampGame3-RampGame1 | -5.603 | 5.124 | -1.094 | 348 | 0.2749 | 0.5321 | 12.768 | 0.285 |
| 1 | 1 | hbo | RampGame4-RampGame1 | -12.371 | 5.201 | -2.378 | 348 | 0.0179 | 0.0861 | 12.962 | 0.281 |
| 1 | 1 | hbr | RampGame4-RampGame2 | 9.507 | 3.040 | 3.128 | 348 | 0.0019 | 0.0176 | 7.574 | 0.481 |
| 1 | 1 | hbr | RampGame2-RampGame1 | 0.169 | 2.827 | 0.060 | 348 | 0.9525 | 0.9896 | 7.044 | 0.517 |
| 1 | 1 | hbr | RampGame3-RampGame2 | 6.445 | 2.817 | 2.288 | 348 | 0.0227 | 0.0975 | 7.020 | 0.518 |
| 1 | 1 | hbr | RampGame4-RampGame3 | 3.062 | 2.761 | 1.109 | 348 | 0.2682 | 0.5277 | 6.881 | 0.529 |
| 1 | 1 | hbr | RampGame3-RampGame1 | 6.614 | 3.167 | 2.088 | 348 | 0.0375 | 0.1407 | 7.893 | 0.461 |
| 1 | 1 | hbr | RampGame4-RampGame1 | 9.676 | 3.170 | 3.052 | 348 | 0.0024 | 0.0217 | 7.899 | 0.461 |
| 1 | 2 | hbo | RampGame4-RampGame2 | -23.060 | 7.091 | -3.252 | 348 | 0.0013 | 0.0136 | 17.670 | 0.206 |
| 1 | 2 | hbo | RampGame2-RampGame1 | 3.390 | 6.477 | 0.523 | 348 | 0.6010 | 0.8189 | 16.141 | 0.225 |
| 1 | 2 | hbo | RampGame3-RampGame2 | -13.448 | 6.719 | -2.002 | 348 | 0.0461 | 0.1555 | 16.743 | 0.217 |
| 1 | 2 | hbo | RampGame4-RampGame3 | -9.612 | 6.482 | -1.483 | 348 | 0.1390 | 0.3627 | 16.153 | 0.225 |
| 1 | 2 | hbo | RampGame3-RampGame1 | -10.058 | 7.057 | -1.425 | 348 | 0.1550 | 0.3795 | 17.585 | 0.207 |
| 1 | 2 | hbo | RampGame4-RampGame1 | -19.670 | 7.248 | -2.714 | 348 | 0.0070 | 0.0446 | 18.061 | 0.202 |
| 1 | 2 | hbr | RampGame4-RampGame2 | 15.096 | 3.828 | 3.944 | 348 | 0.0001 | 0.0019 | 9.539 | 0.382 |
| 1 | 2 | hbr | RampGame2-RampGame1 | 1.171 | 3.469 | 0.338 | 348 | 0.7358 | 0.8781 | 8.644 | 0.421 |
| 1 | 2 | hbr | RampGame3-RampGame2 | 9.102 | 3.468 | 2.625 | 348 | 0.0090 | 0.0538 | 8.641 | 0.421 |
| 1 | 2 | hbr | RampGame4-RampGame3 | 5.994 | 3.465 | 1.730 | 348 | 0.0845 | 0.2504 | 8.634 | 0.422 |
| 1 | 2 | hbr | RampGame3-RampGame1 | 10.273 | 3.921 | 2.620 | 348 | 0.0092 | 0.0538 | 9.772 | 0.372 |
| 1 | 2 | hbr | RampGame4-RampGame1 | 16.267 | 3.952 | 4.116 | 348 | 0.0000 | 0.0012 | 9.849 | 0.370 |
| 2 | 1 | hbo | RampGame4-RampGame2 | -12.491 | 3.550 | -3.519 | 348 | 0.0005 | 0.0079 | 8.846 | 0.411 |
| 2 | 1 | hbo | RampGame2-RampGame1 | -6.918 | 3.355 | -2.062 | 348 | 0.0400 | 0.1476 | 8.361 | 0.435 |
| 2 | 1 | hbo | RampGame3-RampGame2 | -4.705 | 3.368 | -1.397 | 348 | 0.1634 | 0.3844 | 8.394 | 0.434 |
| 2 | 1 | hbo | RampGame4-RampGame3 | -7.786 | 3.380 | -2.304 | 348 | 0.0218 | 0.0952 | 8.422 | 0.432 |
| 2 | 1 | hbo | RampGame3-RampGame1 | -11.622 | 3.581 | -3.245 | 348 | 0.0013 | 0.0136 | 8.925 | 0.408 |
| 2 | 1 | hbo | RampGame4-RampGame1 | -19.408 | 3.571 | -5.435 | 348 | 0.0000 | 0.0000 | 8.898 | 0.409 |
| 2 | 1 | hbr | RampGame4-RampGame2 | 3.044 | 2.125 | 1.432 | 348 | 0.1530 | 0.3795 | 5.296 | 0.687 |
| 2 | 1 | hbr | RampGame2-RampGame1 | -1.661 | 2.005 | -0.829 | 348 | 0.4078 | 0.6750 | 4.995 | 0.729 |
| 2 | 1 | hbr | RampGame3-RampGame2 | 2.673 | 1.998 | 1.337 | 348 | 0.1819 | 0.4159 | 4.980 | 0.731 |
| 2 | 1 | hbr | RampGame4-RampGame3 | 0.371 | 1.993 | 0.186 | 348 | 0.8524 | 0.9281 | 4.966 | 0.733 |
| 2 | 1 | hbr | RampGame3-RampGame1 | 1.012 | 2.196 | 0.461 | 348 | 0.6454 | 0.8277 | 5.473 | 0.665 |
| 2 | 1 | hbr | RampGame4-RampGame1 | 1.383 | 2.227 | 0.621 | 348 | 0.5351 | 0.8122 | 5.550 | 0.656 |
| 2 | 3 | hbo | RampGame4-RampGame2 | -19.805 | 5.220 | -3.794 | 348 | 0.0002 | 0.0032 | 13.009 | 0.280 |
| 2 | 3 | hbo | RampGame2-RampGame1 | -1.563 | 4.833 | -0.323 | 348 | 0.7465 | 0.8781 | 12.044 | 0.302 |
| 2 | 3 | hbo | RampGame3-RampGame2 | -9.542 | 4.911 | -1.943 | 348 | 0.0528 | 0.1669 | 12.238 | 0.297 |
| 2 | 3 | hbo | RampGame4-RampGame3 | -10.264 | 4.883 | -2.102 | 348 | 0.0363 | 0.1405 | 12.169 | 0.299 |
| 2 | 3 | hbo | RampGame3-RampGame1 | -11.105 | 5.279 | -2.104 | 348 | 0.0361 | 0.1405 | 13.155 | 0.277 |
| 2 | 3 | hbo | RampGame4-RampGame1 | -21.369 | 5.305 | -4.028 | 348 | 0.0001 | 0.0015 | 13.220 | 0.275 |
| 2 | 3 | hbr | RampGame4-RampGame2 | 12.582 | 2.797 | 4.498 | 348 | 0.0000 | 0.0004 | 6.971 | 0.522 |
| 2 | 3 | hbr | RampGame2-RampGame1 | -5.436 | 2.671 | -2.035 | 348 | 0.0426 | 0.1504 | 6.657 | 0.547 |
| 2 | 3 | hbr | RampGame3-RampGame2 | 11.324 | 2.644 | 4.283 | 348 | 0.0000 | 0.0007 | 6.589 | 0.552 |
| 2 | 3 | hbr | RampGame4-RampGame3 | 1.258 | 2.642 | 0.476 | 348 | 0.6343 | 0.8228 | 6.584 | 0.553 |
| 2 | 3 | hbr | RampGame3-RampGame1 | 5.888 | 2.947 | 1.998 | 348 | 0.0465 | 0.1555 | 7.345 | 0.496 |
| 2 | 3 | hbr | RampGame4-RampGame1 | 7.146 | 2.949 | 2.423 | 348 | 0.0159 | 0.0795 | 7.349 | 0.495 |
| 3 | 2 | hbo | RampGame4-RampGame2 | -12.031 | 2.932 | -4.103 | 348 | 0.0001 | 0.0012 | 7.307 | 0.498 |
| 3 | 2 | hbo | RampGame2-RampGame1 | -3.789 | 2.656 | -1.427 | 348 | 0.1546 | 0.3795 | 6.618 | 0.550 |
| 3 | 2 | hbo | RampGame3-RampGame2 | -8.609 | 2.671 | -3.223 | 348 | 0.0014 | 0.0136 | 6.657 | 0.547 |
| 3 | 2 | hbo | RampGame4-RampGame3 | -3.422 | 2.724 | -1.256 | 348 | 0.2099 | 0.4538 | 6.789 | 0.536 |
| 3 | 2 | hbo | RampGame3-RampGame1 | -12.398 | 2.897 | -4.280 | 348 | 0.0000 | 0.0007 | 7.219 | 0.504 |
| 3 | 2 | hbo | RampGame4-RampGame1 | -15.820 | 2.934 | -5.392 | 348 | 0.0000 | 0.0000 | 7.311 | 0.498 |
| 3 | 2 | hbr | RampGame4-RampGame2 | 0.863 | 1.611 | 0.536 | 348 | 0.5924 | 0.8189 | 4.015 | 0.907 |
| 3 | 2 | hbr | RampGame2-RampGame1 | 4.877 | 1.482 | 3.292 | 348 | 0.0011 | 0.0136 | 3.692 | 0.986 |
| 3 | 2 | hbr | RampGame3-RampGame2 | 2.813 | 1.461 | 1.926 | 348 | 0.0549 | 0.1711 | 3.640 | 1.000 |
| 3 | 2 | hbr | RampGame4-RampGame3 | -1.950 | 1.499 | -1.301 | 348 | 0.1942 | 0.4396 | 3.735 | 0.974 |
| 3 | 2 | hbr | RampGame3-RampGame1 | 7.690 | 1.613 | 4.767 | 348 | 0.0000 | 0.0002 | 4.020 | 0.905 |
| 3 | 2 | hbr | RampGame4-RampGame1 | 5.740 | 1.632 | 3.517 | 348 | 0.0005 | 0.0079 | 4.067 | 0.895 |
| 3 | 3 | hbo | RampGame4-RampGame2 | -8.604 | 5.858 | -1.469 | 348 | 0.1428 | 0.3685 | 14.598 | 0.249 |
| 3 | 3 | hbo | RampGame2-RampGame1 | -7.591 | 5.314 | -1.428 | 348 | 0.1541 | 0.3795 | 13.242 | 0.275 |
| 3 | 3 | hbo | RampGame3-RampGame2 | -4.371 | 5.274 | -0.829 | 348 | 0.4078 | 0.6750 | 13.143 | 0.277 |
| 3 | 3 | hbo | RampGame4-RampGame3 | -4.233 | 5.374 | -0.788 | 348 | 0.4313 | 0.6995 | 13.391 | 0.272 |
| 3 | 3 | hbo | RampGame3-RampGame1 | -11.962 | 5.828 | -2.053 | 348 | 0.0409 | 0.1486 | 14.523 | 0.251 |
| 3 | 3 | hbo | RampGame4-RampGame1 | -16.195 | 5.906 | -2.742 | 348 | 0.0064 | 0.0428 | 14.717 | 0.247 |
| 3 | 3 | hbr | RampGame4-RampGame2 | 2.231 | 3.330 | 0.670 | 348 | 0.5034 | 0.7948 | 8.298 | 0.439 |
| 3 | 3 | hbr | RampGame2-RampGame1 | 4.472 | 2.976 | 1.502 | 348 | 0.1339 | 0.3531 | 7.416 | 0.491 |
| 3 | 3 | hbr | RampGame3-RampGame2 | 2.362 | 2.910 | 0.812 | 348 | 0.4175 | 0.6817 | 7.252 | 0.502 |
| 3 | 3 | hbr | RampGame4-RampGame3 | -0.132 | 2.971 | -0.044 | 348 | 0.9647 | 0.9945 | 7.404 | 0.492 |
| 3 | 3 | hbr | RampGame3-RampGame1 | 6.834 | 3.351 | 2.039 | 348 | 0.0422 | 0.1504 | 8.350 | 0.436 |
| 3 | 3 | hbr | RampGame4-RampGame1 | 6.702 | 3.396 | 1.974 | 348 | 0.0492 | 0.1596 | 8.463 | 0.430 |
| 3 | 4 | hbo | RampGame4-RampGame2 | -4.752 | 3.439 | -1.382 | 348 | 0.1679 | 0.3913 | 8.570 | 0.425 |
| 3 | 4 | hbo | RampGame2-RampGame1 | 2.961 | 2.965 | 0.999 | 348 | 0.3187 | 0.5791 | 7.388 | 0.493 |
| 3 | 4 | hbo | RampGame3-RampGame2 | -1.188 | 2.964 | -0.401 | 348 | 0.6886 | 0.8608 | 7.385 | 0.493 |
| 3 | 4 | hbo | RampGame4-RampGame3 | -3.563 | 3.067 | -1.162 | 348 | 0.2461 | 0.4963 | 7.642 | 0.476 |
| 3 | 4 | hbo | RampGame3-RampGame1 | 1.772 | 3.354 | 0.528 | 348 | 0.5976 | 0.8189 | 8.359 | 0.435 |
| 3 | 4 | hbo | RampGame4-RampGame1 | -1.791 | 3.421 | -0.523 | 348 | 0.6010 | 0.8189 | 8.525 | 0.427 |
| 3 | 4 | hbr | RampGame4-RampGame2 | -0.911 | 1.732 | -0.526 | 348 | 0.5991 | 0.8189 | 4.316 | 0.843 |
| 3 | 4 | hbr | RampGame2-RampGame1 | -3.466 | 1.736 | -1.997 | 348 | 0.0466 | 0.1555 | 4.325 | 0.841 |
| 3 | 4 | hbr | RampGame3-RampGame2 | -2.431 | 1.702 | -1.428 | 348 | 0.1541 | 0.3795 | 4.241 | 0.858 |
| 3 | 4 | hbr | RampGame4-RampGame3 | 1.520 | 1.782 | 0.853 | 348 | 0.3943 | 0.6665 | 4.441 | 0.820 |
| 3 | 4 | hbr | RampGame3-RampGame1 | -5.897 | 1.826 | -3.229 | 348 | 0.0014 | 0.0136 | 4.551 | 0.800 |
| 3 | 4 | hbr | RampGame4-RampGame1 | -4.377 | 1.785 | -2.452 | 348 | 0.0147 | 0.0750 | 4.448 | 0.818 |
| 4 | 2 | hbo | RampGame4-RampGame2 | 1.669 | 4.832 | 0.345 | 348 | 0.7300 | 0.8781 | 12.041 | 0.302 |
| 4 | 2 | hbo | RampGame2-RampGame1 | 1.394 | 4.309 | 0.323 | 348 | 0.7466 | 0.8781 | 10.738 | 0.339 |
| 4 | 2 | hbo | RampGame3-RampGame2 | 4.288 | 4.289 | 1.000 | 348 | 0.3181 | 0.5791 | 10.687 | 0.341 |
| 4 | 2 | hbo | RampGame4-RampGame3 | -2.619 | 4.429 | -0.591 | 348 | 0.5547 | 0.8130 | 11.036 | 0.330 |
| 4 | 2 | hbo | RampGame3-RampGame1 | 5.682 | 4.707 | 1.207 | 348 | 0.2282 | 0.4805 | 11.730 | 0.310 |
| 4 | 2 | hbo | RampGame4-RampGame1 | 3.063 | 4.753 | 0.644 | 348 | 0.5197 | 0.8027 | 11.843 | 0.307 |
| 4 | 2 | hbr | RampGame4-RampGame2 | -6.167 | 2.617 | -2.356 | 348 | 0.0190 | 0.0895 | 6.523 | 0.558 |
| 4 | 2 | hbr | RampGame2-RampGame1 | -1.574 | 2.649 | -0.594 | 348 | 0.5529 | 0.8130 | 6.601 | 0.551 |
| 4 | 2 | hbr | RampGame3-RampGame2 | -7.721 | 2.569 | -3.005 | 348 | 0.0028 | 0.0236 | 6.403 | 0.568 |
| 4 | 2 | hbr | RampGame4-RampGame3 | 1.554 | 2.703 | 0.575 | 348 | 0.5657 | 0.8149 | 6.736 | 0.540 |
| 4 | 2 | hbr | RampGame3-RampGame1 | -9.295 | 2.741 | -3.391 | 348 | 0.0008 | 0.0117 | 6.831 | 0.533 |
| 4 | 2 | hbr | RampGame4-RampGame1 | -7.741 | 2.636 | -2.937 | 348 | 0.0035 | 0.0265 | 6.568 | 0.554 |
| 4 | 4 | hbo | RampGame4-RampGame2 | -10.112 | 3.904 | -2.590 | 348 | 0.0100 | 0.0572 | 9.730 | 0.374 |
| 4 | 4 | hbo | RampGame2-RampGame1 | 2.343 | 3.550 | 0.660 | 348 | 0.5097 | 0.7995 | 8.847 | 0.411 |
| 4 | 4 | hbo | RampGame3-RampGame2 | -5.861 | 3.557 | -1.648 | 348 | 0.1003 | 0.2865 | 8.863 | 0.411 |
| 4 | 4 | hbo | RampGame4-RampGame3 | -4.251 | 3.603 | -1.180 | 348 | 0.2388 | 0.4941 | 8.977 | 0.405 |
| 4 | 4 | hbo | RampGame3-RampGame1 | -3.518 | 3.878 | -0.907 | 348 | 0.3650 | 0.6302 | 9.664 | 0.377 |
| 4 | 4 | hbo | RampGame4-RampGame1 | -7.769 | 3.850 | -2.018 | 348 | 0.0444 | 0.1544 | 9.595 | 0.379 |
| 4 | 4 | hbr | RampGame4-RampGame2 | 0.047 | 2.489 | 0.019 | 348 | 0.9849 | 0.9949 | 6.202 | 0.587 |
| 4 | 4 | hbr | RampGame2-RampGame1 | -1.182 | 2.277 | -0.519 | 348 | 0.6040 | 0.8189 | 5.674 | 0.641 |
| 4 | 4 | hbr | RampGame3-RampGame2 | 0.858 | 2.299 | 0.373 | 348 | 0.7092 | 0.8781 | 5.730 | 0.635 |
| 4 | 4 | hbr | RampGame4-RampGame3 | -0.811 | 2.311 | -0.351 | 348 | 0.7258 | 0.8781 | 5.758 | 0.632 |
| 4 | 4 | hbr | RampGame3-RampGame1 | -0.324 | 2.449 | -0.132 | 348 | 0.8948 | 0.9587 | 6.102 | 0.596 |
| 4 | 4 | hbr | RampGame4-RampGame1 | -1.135 | 2.475 | -0.459 | 348 | 0.6468 | 0.8277 | 6.168 | 0.590 |
| 4 | 5 | hbo | RampGame4-RampGame2 | 0.189 | 6.819 | 0.028 | 348 | 0.9779 | 0.9945 | 16.993 | 0.214 |
| 4 | 5 | hbo | RampGame2-RampGame1 | -0.856 | 6.311 | -0.136 | 348 | 0.8922 | 0.9587 | 15.726 | 0.231 |
| 4 | 5 | hbo | RampGame3-RampGame2 | 3.020 | 6.317 | 0.478 | 348 | 0.6329 | 0.8228 | 15.741 | 0.231 |
| 4 | 5 | hbo | RampGame4-RampGame3 | -2.831 | 6.331 | -0.447 | 348 | 0.6550 | 0.8277 | 15.776 | 0.231 |
| 4 | 5 | hbo | RampGame3-RampGame1 | 2.164 | 6.822 | 0.317 | 348 | 0.7512 | 0.8781 | 16.999 | 0.214 |
| 4 | 5 | hbo | RampGame4-RampGame1 | -0.667 | 6.853 | -0.097 | 348 | 0.9225 | 0.9754 | 17.077 | 0.213 |
| 4 | 5 | hbr | RampGame4-RampGame2 | 2.106 | 4.185 | 0.503 | 348 | 0.6151 | 0.8228 | 10.429 | 0.349 |
| 4 | 5 | hbr | RampGame2-RampGame1 | -3.498 | 3.835 | -0.912 | 348 | 0.3624 | 0.6302 | 9.557 | 0.381 |
| 4 | 5 | hbr | RampGame3-RampGame2 | -1.123 | 3.849 | -0.292 | 348 | 0.7706 | 0.8854 | 9.592 | 0.379 |
| 4 | 5 | hbr | RampGame4-RampGame3 | 3.230 | 3.828 | 0.844 | 348 | 0.3995 | 0.6704 | 9.540 | 0.382 |
| 4 | 5 | hbr | RampGame3-RampGame1 | -4.621 | 4.188 | -1.104 | 348 | 0.2706 | 0.5279 | 10.435 | 0.349 |
| 4 | 5 | hbr | RampGame4-RampGame1 | -1.391 | 4.225 | -0.329 | 348 | 0.7421 | 0.8781 | 10.530 | 0.346 |
| 5 | 3 | hbo | RampGame4-RampGame2 | -2.770 | 3.120 | -0.888 | 348 | 0.3752 | 0.6387 | 7.775 | 0.468 |
| 5 | 3 | hbo | RampGame2-RampGame1 | 3.450 | 2.701 | 1.278 | 348 | 0.2022 | 0.4453 | 6.730 | 0.541 |
| 5 | 3 | hbo | RampGame3-RampGame2 | -1.533 | 2.762 | -0.555 | 348 | 0.5793 | 0.8188 | 6.883 | 0.529 |
| 5 | 3 | hbo | RampGame4-RampGame3 | -1.237 | 2.769 | -0.447 | 348 | 0.6553 | 0.8277 | 6.900 | 0.527 |
| 5 | 3 | hbo | RampGame3-RampGame1 | 1.918 | 3.112 | 0.616 | 348 | 0.5381 | 0.8122 | 7.754 | 0.469 |
| 5 | 3 | hbo | RampGame4-RampGame1 | 0.680 | 3.120 | 0.218 | 348 | 0.8275 | 0.9195 | 7.775 | 0.468 |
| 5 | 3 | hbr | RampGame4-RampGame2 | 3.088 | 1.962 | 1.574 | 348 | 0.1164 | 0.3211 | 4.889 | 0.745 |
| 5 | 3 | hbr | RampGame2-RampGame1 | -2.485 | 1.760 | -1.412 | 348 | 0.1590 | 0.3815 | 4.387 | 0.830 |
| 5 | 3 | hbr | RampGame3-RampGame2 | 3.448 | 1.772 | 1.946 | 348 | 0.0525 | 0.1669 | 4.415 | 0.824 |
| 5 | 3 | hbr | RampGame4-RampGame3 | -0.360 | 1.761 | -0.204 | 348 | 0.8381 | 0.9203 | 4.387 | 0.830 |
| 5 | 3 | hbr | RampGame3-RampGame1 | 0.963 | 1.976 | 0.487 | 348 | 0.6264 | 0.8228 | 4.923 | 0.739 |
| 5 | 3 | hbr | RampGame4-RampGame1 | 0.603 | 2.000 | 0.301 | 348 | 0.7634 | 0.8850 | 4.985 | 0.730 |
| 5 | 4 | hbo | RampGame4-RampGame2 | -0.575 | 3.135 | -0.183 | 348 | 0.8546 | 0.9281 | 7.813 | 0.466 |
| 5 | 4 | hbo | RampGame2-RampGame1 | 0.671 | 2.673 | 0.251 | 348 | 0.8019 | 0.9079 | 6.662 | 0.546 |
| 5 | 4 | hbo | RampGame3-RampGame2 | -0.861 | 2.741 | -0.314 | 348 | 0.7537 | 0.8781 | 6.831 | 0.533 |
| 5 | 4 | hbo | RampGame4-RampGame3 | 0.286 | 2.758 | 0.104 | 348 | 0.9175 | 0.9743 | 6.872 | 0.530 |
| 5 | 4 | hbo | RampGame3-RampGame1 | -0.190 | 3.094 | -0.061 | 348 | 0.9511 | 0.9896 | 7.711 | 0.472 |
| 5 | 4 | hbo | RampGame4-RampGame1 | 0.096 | 3.121 | 0.031 | 348 | 0.9755 | 0.9945 | 7.777 | 0.468 |
| 5 | 4 | hbr | RampGame4-RampGame2 | 4.817 | 1.929 | 2.498 | 348 | 0.0130 | 0.0707 | 4.806 | 0.757 |
| 5 | 4 | hbr | RampGame2-RampGame1 | -1.713 | 1.719 | -0.996 | 348 | 0.3197 | 0.5791 | 4.284 | 0.849 |
| 5 | 4 | hbr | RampGame3-RampGame2 | 1.565 | 1.717 | 0.911 | 348 | 0.3627 | 0.6302 | 4.278 | 0.851 |
| 5 | 4 | hbr | RampGame4-RampGame3 | 3.253 | 1.712 | 1.900 | 348 | 0.0582 | 0.1792 | 4.265 | 0.853 |
| 5 | 4 | hbr | RampGame3-RampGame1 | -0.148 | 1.947 | -0.076 | 348 | 0.9393 | 0.9887 | 4.851 | 0.750 |
| 5 | 4 | hbr | RampGame4-RampGame1 | 3.104 | 1.969 | 1.576 | 348 | 0.1159 | 0.3211 | 4.908 | 0.742 |
| 5 | 6 | hbo | RampGame4-RampGame2 | -10.964 | 3.269 | -3.353 | 348 | 0.0009 | 0.0118 | 8.147 | 0.447 |
| 5 | 6 | hbo | RampGame2-RampGame1 | 1.467 | 2.922 | 0.502 | 348 | 0.6161 | 0.8228 | 7.282 | 0.500 |
| 5 | 6 | hbo | RampGame3-RampGame2 | -0.964 | 2.965 | -0.325 | 348 | 0.7454 | 0.8781 | 7.389 | 0.493 |
| 5 | 6 | hbo | RampGame4-RampGame3 | -10.000 | 2.970 | -3.367 | 348 | 0.0008 | 0.0118 | 7.400 | 0.492 |
| 5 | 6 | hbo | RampGame3-RampGame1 | 0.503 | 3.271 | 0.154 | 348 | 0.8779 | 0.9491 | 8.151 | 0.447 |
| 5 | 6 | hbo | RampGame4-RampGame1 | -9.497 | 3.344 | -2.840 | 348 | 0.0048 | 0.0339 | 8.334 | 0.437 |
| 5 | 6 | hbr | RampGame4-RampGame2 | 7.203 | 2.658 | 2.710 | 348 | 0.0071 | 0.0446 | 6.623 | 0.550 |
| 5 | 6 | hbr | RampGame2-RampGame1 | 6.533 | 2.357 | 2.771 | 348 | 0.0059 | 0.0403 | 5.874 | 0.620 |
| 5 | 6 | hbr | RampGame3-RampGame2 | 0.088 | 2.380 | 0.037 | 348 | 0.9705 | 0.9945 | 5.930 | 0.614 |
| 5 | 6 | hbr | RampGame4-RampGame3 | 7.114 | 2.406 | 2.957 | 348 | 0.0033 | 0.0257 | 5.996 | 0.607 |
| 5 | 6 | hbr | RampGame3-RampGame1 | 6.621 | 2.659 | 2.490 | 348 | 0.0132 | 0.0707 | 6.627 | 0.549 |
| 5 | 6 | hbr | RampGame4-RampGame1 | 13.735 | 2.684 | 5.117 | 348 | 0.0000 | 0.0000 | 6.689 | 0.544 |
| 6 | 4 | hbo | RampGame4-RampGame2 | -6.723 | 3.047 | -2.206 | 348 | 0.0280 | 0.1140 | 7.593 | 0.479 |
| 6 | 4 | hbo | RampGame2-RampGame1 | -1.675 | 2.830 | -0.592 | 348 | 0.5543 | 0.8130 | 7.051 | 0.516 |
| 6 | 4 | hbo | RampGame3-RampGame2 | -1.680 | 2.864 | -0.587 | 348 | 0.5579 | 0.8130 | 7.136 | 0.510 |
| 6 | 4 | hbo | RampGame4-RampGame3 | -5.043 | 2.847 | -1.771 | 348 | 0.0774 | 0.2351 | 7.095 | 0.513 |
| 6 | 4 | hbo | RampGame3-RampGame1 | -3.355 | 3.137 | -1.069 | 348 | 0.2857 | 0.5485 | 7.818 | 0.466 |
| 6 | 4 | hbo | RampGame4-RampGame1 | -8.398 | 3.146 | -2.669 | 348 | 0.0080 | 0.0490 | 7.841 | 0.464 |
| 6 | 4 | hbr | RampGame4-RampGame2 | 5.500 | 2.461 | 2.235 | 348 | 0.0261 | 0.1098 | 6.133 | 0.593 |
| 6 | 4 | hbr | RampGame2-RampGame1 | 2.428 | 2.172 | 1.117 | 348 | 0.2646 | 0.5248 | 5.413 | 0.672 |
| 6 | 4 | hbr | RampGame3-RampGame2 | 0.004 | 2.198 | 0.002 | 348 | 0.9987 | 0.9987 | 5.477 | 0.664 |
| 6 | 4 | hbr | RampGame4-RampGame3 | 5.496 | 2.220 | 2.476 | 348 | 0.0138 | 0.0718 | 5.532 | 0.658 |
| 6 | 4 | hbr | RampGame3-RampGame1 | 2.431 | 2.446 | 0.994 | 348 | 0.3209 | 0.5791 | 6.095 | 0.597 |
| 6 | 4 | hbr | RampGame4-RampGame1 | 7.927 | 2.464 | 3.217 | 348 | 0.0014 | 0.0136 | 6.141 | 0.593 |
| 6 | 5 | hbo | RampGame4-RampGame2 | -4.985 | 2.510 | -1.986 | 348 | 0.0478 | 0.1573 | 6.256 | 0.582 |
| 6 | 5 | hbo | RampGame2-RampGame1 | 1.242 | 2.242 | 0.554 | 348 | 0.5800 | 0.8188 | 5.586 | 0.652 |
| 6 | 5 | hbo | RampGame3-RampGame2 | -2.946 | 2.169 | -1.358 | 348 | 0.1753 | 0.4045 | 5.406 | 0.673 |
| 6 | 5 | hbo | RampGame4-RampGame3 | -2.039 | 2.280 | -0.894 | 348 | 0.3719 | 0.6375 | 5.683 | 0.640 |
| 6 | 5 | hbo | RampGame3-RampGame1 | -1.704 | 2.510 | -0.679 | 348 | 0.4976 | 0.7909 | 6.256 | 0.582 |
| 6 | 5 | hbo | RampGame4-RampGame1 | -3.743 | 2.485 | -1.506 | 348 | 0.1329 | 0.3531 | 6.192 | 0.588 |
| 6 | 5 | hbr | RampGame4-RampGame2 | 1.946 | 1.657 | 1.174 | 348 | 0.2410 | 0.4944 | 4.130 | 0.881 |
| 6 | 5 | hbr | RampGame2-RampGame1 | 0.864 | 1.650 | 0.524 | 348 | 0.6008 | 0.8189 | 4.112 | 0.885 |
| 6 | 5 | hbr | RampGame3-RampGame2 | 1.965 | 1.595 | 1.232 | 348 | 0.2187 | 0.4687 | 3.975 | 0.916 |
| 6 | 5 | hbr | RampGame4-RampGame3 | -0.019 | 1.634 | -0.012 | 348 | 0.9907 | 0.9949 | 4.073 | 0.894 |
| 6 | 5 | hbr | RampGame3-RampGame1 | 2.830 | 1.703 | 1.661 | 348 | 0.0975 | 0.2820 | 4.244 | 0.858 |
| 6 | 5 | hbr | RampGame4-RampGame1 | 2.811 | 1.675 | 1.678 | 348 | 0.0943 | 0.2761 | 4.175 | 0.872 |
| 6 | 6 | hbo | RampGame4-RampGame2 | -9.611 | 4.124 | -2.330 | 348 | 0.0204 | 0.0935 | 10.278 | 0.354 |
| 6 | 6 | hbo | RampGame2-RampGame1 | 0.058 | 3.717 | 0.016 | 348 | 0.9876 | 0.9949 | 9.262 | 0.393 |
| 6 | 6 | hbo | RampGame3-RampGame2 | -5.355 | 3.561 | -1.504 | 348 | 0.1335 | 0.3531 | 8.875 | 0.410 |
| 6 | 6 | hbo | RampGame4-RampGame3 | -4.256 | 3.728 | -1.142 | 348 | 0.2544 | 0.5088 | 9.289 | 0.392 |
| 6 | 6 | hbo | RampGame3-RampGame1 | -5.298 | 4.164 | -1.272 | 348 | 0.2042 | 0.4455 | 10.377 | 0.351 |
| 6 | 6 | hbo | RampGame4-RampGame1 | -9.553 | 4.109 | -2.325 | 348 | 0.0206 | 0.0935 | 10.239 | 0.355 |
| 6 | 6 | hbr | RampGame4-RampGame2 | -0.807 | 2.772 | -0.291 | 348 | 0.7710 | 0.8854 | 6.908 | 0.527 |
| 6 | 6 | hbr | RampGame2-RampGame1 | 2.427 | 2.667 | 0.910 | 348 | 0.3633 | 0.6302 | 6.645 | 0.548 |
| 6 | 6 | hbr | RampGame3-RampGame2 | 0.543 | 2.681 | 0.202 | 348 | 0.8398 | 0.9203 | 6.682 | 0.545 |
| 6 | 6 | hbr | RampGame4-RampGame3 | -1.350 | 2.699 | -0.500 | 348 | 0.6173 | 0.8228 | 6.727 | 0.541 |
| 6 | 6 | hbr | RampGame3-RampGame1 | 2.970 | 2.819 | 1.054 | 348 | 0.2928 | 0.5577 | 7.024 | 0.518 |
| 6 | 6 | hbr | RampGame4-RampGame1 | 1.620 | 2.827 | 0.573 | 348 | 0.5671 | 0.8149 | 7.045 | 0.517 |
| 7 | 5 | hbo | RampGame4-RampGame2 | -2.437 | 4.165 | -0.585 | 348 | 0.5589 | 0.8130 | 10.379 | 0.351 |
| 7 | 5 | hbo | RampGame2-RampGame1 | 1.068 | 3.901 | 0.274 | 348 | 0.7843 | 0.8921 | 9.720 | 0.374 |
| 7 | 5 | hbo | RampGame3-RampGame2 | 1.604 | 3.897 | 0.412 | 348 | 0.6808 | 0.8555 | 9.711 | 0.375 |
| 7 | 5 | hbo | RampGame4-RampGame3 | -4.041 | 3.982 | -1.015 | 348 | 0.3109 | 0.5785 | 9.924 | 0.367 |
| 7 | 5 | hbo | RampGame3-RampGame1 | 2.673 | 4.189 | 0.638 | 348 | 0.5239 | 0.8027 | 10.440 | 0.349 |
| 7 | 5 | hbo | RampGame4-RampGame1 | -1.368 | 4.180 | -0.327 | 348 | 0.7436 | 0.8781 | 10.417 | 0.349 |
| 7 | 5 | hbr | RampGame4-RampGame2 | -1.533 | 3.178 | -0.483 | 348 | 0.6298 | 0.8228 | 7.919 | 0.460 |
| 7 | 5 | hbr | RampGame2-RampGame1 | -0.909 | 3.241 | -0.280 | 348 | 0.7793 | 0.8907 | 8.077 | 0.451 |
| 7 | 5 | hbr | RampGame3-RampGame2 | -3.310 | 3.181 | -1.041 | 348 | 0.2988 | 0.5646 | 7.927 | 0.459 |
| 7 | 5 | hbr | RampGame4-RampGame3 | 1.777 | 3.180 | 0.559 | 348 | 0.5766 | 0.8188 | 7.924 | 0.459 |
| 7 | 5 | hbr | RampGame3-RampGame1 | -4.219 | 3.299 | -1.279 | 348 | 0.2018 | 0.4453 | 8.221 | 0.443 |
| 7 | 5 | hbr | RampGame4-RampGame1 | -2.442 | 3.319 | -0.736 | 348 | 0.4623 | 0.7398 | 8.271 | 0.440 |
| 7 | 7 | hbo | RampGame4-RampGame2 | 0.386 | 5.726 | 0.067 | 348 | 0.9462 | 0.9896 | 14.268 | 0.255 |
| 7 | 7 | hbo | RampGame2-RampGame1 | 11.718 | 5.085 | 2.305 | 348 | 0.0218 | 0.0952 | 12.670 | 0.287 |
| 7 | 7 | hbo | RampGame3-RampGame2 | 1.573 | 4.975 | 0.316 | 348 | 0.7521 | 0.8781 | 12.398 | 0.294 |
| 7 | 7 | hbo | RampGame4-RampGame3 | -1.187 | 5.184 | -0.229 | 348 | 0.8191 | 0.9186 | 12.917 | 0.282 |
| 7 | 7 | hbo | RampGame3-RampGame1 | 13.290 | 5.589 | 2.378 | 348 | 0.0179 | 0.0861 | 13.927 | 0.261 |
| 7 | 7 | hbo | RampGame4-RampGame1 | 12.104 | 5.743 | 2.108 | 348 | 0.0358 | 0.1405 | 14.311 | 0.254 |
| 7 | 7 | hbr | RampGame4-RampGame2 | 5.468 | 4.681 | 1.168 | 348 | 0.2435 | 0.4953 | 11.664 | 0.312 |
| 7 | 7 | hbr | RampGame2-RampGame1 | -6.588 | 4.382 | -1.504 | 348 | 0.1336 | 0.3531 | 10.919 | 0.333 |
| 7 | 7 | hbr | RampGame3-RampGame2 | 0.952 | 4.290 | 0.222 | 348 | 0.8245 | 0.9195 | 10.691 | 0.340 |
| 7 | 7 | hbr | RampGame4-RampGame3 | 4.516 | 4.367 | 1.034 | 348 | 0.3018 | 0.5658 | 10.882 | 0.334 |
| 7 | 7 | hbr | RampGame3-RampGame1 | -5.636 | 4.612 | -1.222 | 348 | 0.2225 | 0.4725 | 11.492 | 0.317 |
| 7 | 7 | hbr | RampGame4-RampGame1 | -1.120 | 4.583 | -0.244 | 348 | 0.8071 | 0.9094 | 11.420 | 0.319 |
| 8 | 6 | hbo | RampGame4-RampGame2 | -0.402 | 3.804 | -0.106 | 348 | 0.9159 | 0.9743 | 9.479 | 0.384 |
| 8 | 6 | hbo | RampGame2-RampGame1 | -2.126 | 3.342 | -0.636 | 348 | 0.5251 | 0.8027 | 8.328 | 0.437 |
| 8 | 6 | hbo | RampGame3-RampGame2 | -1.558 | 3.264 | -0.477 | 348 | 0.6334 | 0.8228 | 8.133 | 0.448 |
| 8 | 6 | hbo | RampGame4-RampGame3 | 1.156 | 3.385 | 0.341 | 348 | 0.7329 | 0.8781 | 8.435 | 0.432 |
| 8 | 6 | hbo | RampGame3-RampGame1 | -3.684 | 3.805 | -0.968 | 348 | 0.3336 | 0.5976 | 9.482 | 0.384 |
| 8 | 6 | hbo | RampGame4-RampGame1 | -2.528 | 3.867 | -0.654 | 348 | 0.5137 | 0.8005 | 9.636 | 0.378 |
| 8 | 6 | hbr | RampGame4-RampGame2 | -6.664 | 2.582 | -2.581 | 348 | 0.0103 | 0.0572 | 6.434 | 0.566 |
| 8 | 6 | hbr | RampGame2-RampGame1 | -4.300 | 2.469 | -1.742 | 348 | 0.0824 | 0.2472 | 6.151 | 0.592 |
| 8 | 6 | hbr | RampGame3-RampGame2 | -3.420 | 2.440 | -1.402 | 348 | 0.1619 | 0.3844 | 6.079 | 0.599 |
| 8 | 6 | hbr | RampGame4-RampGame3 | -3.244 | 2.527 | -1.284 | 348 | 0.1999 | 0.4453 | 6.296 | 0.578 |
| 8 | 6 | hbr | RampGame3-RampGame1 | -7.720 | 2.557 | -3.019 | 348 | 0.0027 | 0.0234 | 6.373 | 0.571 |
| 8 | 6 | hbr | RampGame4-RampGame1 | -10.964 | 2.551 | -4.297 | 348 | 0.0000 | 0.0007 | 6.358 | 0.572 |
| 8 | 7 | hbo | RampGame4-RampGame2 | 2.477 | 4.061 | 0.610 | 348 | 0.5423 | 0.8130 | 10.121 | 0.360 |
| 8 | 7 | hbo | RampGame2-RampGame1 | -2.627 | 3.505 | -0.750 | 348 | 0.4540 | 0.7313 | 8.734 | 0.417 |
| 8 | 7 | hbo | RampGame3-RampGame2 | -0.687 | 3.370 | -0.204 | 348 | 0.8385 | 0.9203 | 8.397 | 0.433 |
| 8 | 7 | hbo | RampGame4-RampGame3 | 3.164 | 3.481 | 0.909 | 348 | 0.3640 | 0.6302 | 8.674 | 0.420 |
| 8 | 7 | hbo | RampGame3-RampGame1 | -3.315 | 4.073 | -0.814 | 348 | 0.4163 | 0.6817 | 10.149 | 0.359 |
| 8 | 7 | hbo | RampGame4-RampGame1 | -0.150 | 4.134 | -0.036 | 348 | 0.9710 | 0.9945 | 10.302 | 0.353 |
| 8 | 7 | hbr | RampGame4-RampGame2 | -5.592 | 2.535 | -2.206 | 348 | 0.0280 | 0.1140 | 6.316 | 0.576 |
| 8 | 7 | hbr | RampGame2-RampGame1 | -2.703 | 2.253 | -1.200 | 348 | 0.2311 | 0.4823 | 5.614 | 0.648 |
| 8 | 7 | hbr | RampGame3-RampGame2 | -4.754 | 2.274 | -2.091 | 348 | 0.0373 | 0.1407 | 5.666 | 0.642 |
| 8 | 7 | hbr | RampGame4-RampGame3 | -0.838 | 2.361 | -0.355 | 348 | 0.7228 | 0.8781 | 5.885 | 0.618 |
| 8 | 7 | hbr | RampGame3-RampGame1 | -7.457 | 2.507 | -2.974 | 348 | 0.0031 | 0.0252 | 6.248 | 0.583 |
| 8 | 7 | hbr | RampGame4-RampGame1 | -8.295 | 2.543 | -3.262 | 348 | 0.0012 | 0.0136 | 6.336 | 0.574 |

# Supplementary ECG results

MD indicates mean difference.

## Time domain variables

For MeanRR [ms] the custom contrasts revealed no significant differences: Ramp2-Ramp1 (MD = -8.7, 95%CI [-0.8, 18.1], p = 0.071, η^2^ = 0.130), Hard1 and Easy1 (MD =0.8, 95%CI [-26.1, 27.7], p = 0.951, η^2^ = 0.000), or between Easy2 and Easy1 (MD = 8.4, 95%CI [-2.5, 19.4], p = 0.125, η^2^ = 0.095), or between Hard2 and Hard1(MD = 17.4, 95%CI [1.8, 33.0], p = 0.030, η^2^ = 0.181).

For SDNN [ms] the custom contrasts revealed no significant differences: Ramp2-Ramp1 (MD = -3.9, 95%CI [-10.0, 2.2], p = 0.200, η^2^ = 0.067), Hard1-Easy1 (MD = -1.6, 95%CI [-5.8, 2.5], p = 0.424, η^2^ = 0.027), Easy2-Easy1 (MD = -2.3, 95%CI [-6.2, 1.5], p = 0.221, η^2^ = 0.062), Hard2-Hard1 (MD = 2.0, 95%CI [-3.9, 8.0], p = 0.486, η^2^ = 0.020).

For MeanHR [bpm] the custom contrasts revealed no significant differences for all contrasts: Ramp2-Ramp1 (MD = -0.5, 95%CI [-1.9, 0.9], p = 0.342, η^2^ = 0.038), Hard1-Easy1 (MD = 0.6, 95%CI [-3.7, 5.0], p = 0.697, η^2^ = 0.006), Easy2-Easy1 (MD = -1.1, 95%CI [-2.8, 0.6], p = 0.093, η^2^ = 0.113), and Hard2-Hard1 (MD = -2.1, 95%CI [-4.6, 0.4], p = 0.025, η^2^ = 0.193).

For SDHR [bpm] the custom contrasts revealed no significant differences: Ramp2-Ramp1 (MD = -0.5, 95%CI [-1.2, 0.2], p = 0.151, η^2^ = 0.084), Hard1-Easy1 (MD = -0.2, 95%CI [-0.6, 0.2], p = 0.293, η^2^ = 0.046), Easy2-Easy1 (MD = -0.4, 95%CI [-0.8, -0.003], p = 0.048, η^2^ = 0.153), and Hard2-Hard1 (MD = 0.043, 95%CI [-0.481, 0.567], p = 0.868, η^2^ = 0.001).

For RMSSD [ms] the custom contrasts revealed no significant differences: Ramp2-Ramp1 (MD = -8.5, 95%CI [-23.9, 6.9], p = 0.265, η^2^ = 0.051), Hard1-Easy1 (MD= 0.4, 95%CI [-4.3, 5.2], p = 0.862, η^2^ = 0.001), Easy2-Easy1 (MD = -2.1, 95%CI [-8.0, 3.8], p = 0.476, η^2^ = 0.021), and Hard2-Hard1(MD = -1.8, 95%CI [-12.8, 9.3], p = 0.745, η^2^ = 0.004).

For pNN20 [%] the custom contrast revealed no significant differences: Ramp2-Ramp1 (MD = -0.1, 95%CI [-2.4, 2.6], p = 0.920, η^2^ < 0.001), Hard1-Easy1 (MD = -1.7, 95%CI [-6.0, 2.7], p = 0.443, η^2^ = 0.025), Easy2-Easy1 (MD = -0.1, 95%CI [-2.0, 1.7], p = 0.885, η^2^ = 0.001), and Hard2-Hard1 (MD = 2.9, 95%CI [-0.1, 5.9], p = 0.060, η^2^ = 0.140).

## Frequency domain variables

For LFpeak [Hz] the custom contrasts revealed no significant differences: Ramp2-Ramp1 (MD = -0.001, 95%CI [-0.005, 0.007], p = 0.856, η^2^ = 0.001), Hard1-Easy1 (MD = 0.002, 95%CI [-0.004, 0.008], p = 0.456, η^2^ = 0.023), Easy2-Easy1 (MD < 0.001, 95%CI [-0.007, 0.007], p = 0.938, η^2^ < 0.001), and Hard2-Hard1 (MD < 0.001, 95%CI [-0.006, 0.006], p = 0.964, η^2^ < 0.001). There were no significant differences for LFpeak.

For LFpow [ms^2^] the custom contrasts revealed no significant differences: Ramp2-Ramp1 (MD = 127.8, 95%CI [-407.6, 663.2], p = 0.627, η^2^ = 0.010), Hard1-Easy1 (MD = -115.5, 95%CI [-340.4, 109.3], p = 0.300, η^2^ = 0.045), Easy2-Easy1 (MD = -125.5, 95%CI [-343.9, 93.0], p = 0.247, η^2^ = 0.055), and Hard2-Hard1 (MD = 243.8, 95%CI [43.6, 444.0], p = 0.019, η^2^ = 0.208).

For HFpow [ms2] the custom contrasts yielded no significant differences: Ramp2-Ramp1 (MD = -556.3, 95%CI [-1517.9, 405.3], p = 0.244, η^2^ = 0.056), Hard1-Easy1 (MD = 119.4, 95%CI [-184.2, 423.0], p = 0.425, η^2^ = 0.027), Easy2-Easy1 (MD = -188.2, 95%CI [-558.5, 182.0], p = 0.304, η^2^ = 0.044), and Hard2-Hard1 (MD = -257.6, 95%CI [-1052.8, 537.6], p = 0.510, η^2^ = 0.018).

LFpow [log] the custom contrasts revealed no significant differences for all contrasts: Ramp2-Ramp1 (MD = -0.02, 95%CI [-0.27, 0.30], p = 0.906, η^2^ = 0.0.001), Hard1-Easy1 (MD = -0.16, 95%CI [-0.44, 0.11], p = 0.235, η^2^ =0 .058), Easy2-Easy1 (MD = -0.16, 95%CI [-0.41, 0.10], p = 0.218, η^2^ = 0.063), and Hard2-Hard1 (MD = 0.18, 95%CI [-0.11, 0.48], p = 0.214, η^2^ = 0.064).

For LFpow [%] The custom contrasts revealed no significant differences for all contrasts: Ramp2-Ramp1 (MD = 5.2, 95%CI [-2.4, 12.8], p = 0.171, η^2^ = 0.076). Remaining contrasts were insignificant: Hard1-Easy1 (MD = -1.6, 95%CI [-5.8, 2.6], p = 0.445, η^2^ = 0.024), Easy2-Easy1 (MD = -1.2, 95%CI [-6.3, 4.0], p = 0.645, η^2^ = 0.009), and Hard2-Hard1 (MD = 1.6, 95%CI [-4.1, 7.3], p = 0.561, η^2^ = 0.014).

For HFpow [%] the custom contrasts revealed no significant differences for all contrasts: Ramp2-Ramp1 (MD = -5.6, 95%CI [-13.1, 1.9], p = 0.134, η^2^ = 0.091), Hard1-Easy1 (MD = 2.1, 95%CI [-1.4, 5.5], p = 0.237, η^2^ = 0.058), Easy2-Easy1 (MD = 1.4, 95%CI [-3.6, 6.4], p = 0.571, η^2^ = 0.014), and Hard2-Hard1 (MD = -1.5, 95%CI [-6.9, 4.0], p = 0.584, η^2^ = 0.013).

For LFpow [n.u.] the custom contrasts revealed no significant differences for all contrasts: Ramp2-Ramp1 (MD = 5.8, 95%CI [-2.0, 13.6], p = 0.135, η^2^ = 0.091), Hard1-Easy1 (MD = -2.1, 95%CI [-6.1, 1.8], p = 0.274, η^2^ = 0.050), Easy2-Easy1 (MD = -1.5, 95%CI [-6.8, 3.8], p = 0.561, η^2^ = 0.014), and Hard2-Hard1 (MD = 1.6, 95%CI [-4.3, 7.5], p = 0.585, η^2^ = 0.013).

For HFpow [n.u.] the custom contrasts revealed no significant differences for all contrasts: Ramp2-Ramp1 (MD = -5.9, 95%CI [-13.6, 1.9], p = 0.132, η^2^ = 0.092). Remaining contrasts were insignificant: Hard1-Easy1 (MD = 2.1, 95%CI [-1.8, 6.0], p = 0.269, η^2^ = 0.051), Easy2-Easy1 (MD = 1.5, 95%CI [-3.7, 6.8], p = 0.551, η^2^ = 0.015), and Hard2-Hard1 (MD = -1.5, 95%CI [-7.3, 4.3], p = 0.598, η^2^ = 0.012).

For LF_HF_ratio the custom contrasts revealed no significant differences for all contrasts: Ramp2-Ramp1 (MD = 0.44, 95%CI [-0.31, 1.20], p = 0.239, η^2^ = 0.057), Hard1-Easy1 (MD = 0.13, 95%CI [-0.63, 0.89], p = 0.729, η^2^ = 0.005), Easy2-Easy1 (MD = -0.24, 5%CI [-0.80, 0.32], p = 0.387, η^2^ = 0.031), and Hard2-Hard1 (MD = 0.12, 95%CI [-0.67, 0.91], p = 0.751, η^2^ = 0.004).

# Generation of pseudorandom alarm intervals

The pseudorandom alarm timings were generated using the [RandomInteger](https://reference.wolfram.com/language/ref/RandomInteger.html) function from Wolfram Language between 5 and 235 seconds (i.e., not in the first or last 5 seconds of the task). After number generation we ensured that the time between two alarms should be more than 5 seconds, and that the timings were somewhat distributed across task duration (i.e., not all alarms exclusively at the start or end of the task). Because these timings are irregular without a consistent interval, our assumption was that the participant will experience these timings as random.

# Further explanation of Tetris parameters

The level refers to the level of difficulty. The speed is defined as the delay (in seconds) between each incremental downward movement of the tetromino. The level of difficulty and downward speed is governed by the following equation:

$${speed=baseSpeed}^{(1+\frac{level}{10})}$$

Base speed is constant at 0.4 seconds. Thus, for level one, the tetrominoes move one row down every 365 ms, whereas for level 12, the tetrominoes move one row down every 133 ms.

Increments refers to the number of evenly distributed levels of difficulty. I.e., Practice has the following level 1,2,3,4,5, Ramp has the following levels 1,3,6,9,12,15.

# References

Santosa, H., Zhai, X., Fishburn, F., Huppert, T., 2018. The NIRS Brain AnalyzIR Toolbox. Algorithms 11, 73. https://doi.org/10.3390/a11050073
